# Association of Sleep-Related Hypoxia With Risk of COVID-19 Hospitalizations and Mortality in a Large Integrated Health System

**DOI:** 10.1001/jamanetworkopen.2021.34241

**Published:** 2021-11-10

**Authors:** Cinthya Pena Orbea, Lu Wang, Vaishal Shah, Lara Jehi, Alex Milinovich, Nancy Foldvary-Schaefer, Mina K. Chung, Saif Mashaqi, Loutfi Aboussouan, Kelsey Seidel, Reena Mehra

**Affiliations:** 1 Sleep Disorders Center, Neurological Institute, Cleveland Clinic, Cleveland, Ohio; 2 Quantitative Health Science Department, Cleveland Clinic, Cleveland, Ohio; 3 Neurological Institute, Cleveland Clinic, Cleveland, Ohio; 4 Heart, Vascular and Thoracic Institute, Department of Cardiovascular Medicine, Cleveland Clinic, Cleveland, Ohio; 5 Department of Pulmonary, Critical Care and Sleep Medicine, University of Arizona School of Medicine, Tucson; 6 Respiratory Institute, Heart and Vascular Institute and Lerner Research Institute, Cleveland Clinic, Cleveland, Ohio; 7 Department of Pharmacy Practice, Northeast Ohio Medical University, Rootstown

## Abstract

**Importance:**

The influence of sleep-disordered breathing (SDB) and sleep-related hypoxemia in SARS-CoV-2 viral infection and COVID-19 outcomes remains unknown. Controversy exists regarding whether to continue treatment for SDB with positive airway pressure given concern for aerosolization with limited data to inform professional society recommendations.

**Objective:**

To investigate the association of SDB (identified via polysomnogram) and sleep-related hypoxia with (1) SARS-CoV-2 positivity and (2) World Health Organization (WHO)-designated COVID-19 clinical outcomes while accounting for confounding including obesity, underlying cardiopulmonary disease, cancer, and smoking history.

**Design, Setting, and Participants:**

This case-control study was conducted within the Cleveland Clinic Health System (Ohio and Florida) and included all patients who were tested for COVID-19 between March 8 and November 30, 2020, and who had an available sleep study record. Sleep indices and SARS-CoV-2 positivity were assessed with overlap propensity score weighting, and COVID-19 clinical outcomes were assessed using the institutional registry.

**Exposures:**

Sleep study–identified SDB (defined by frequency of apneas and hypopneas using the Apnea-Hypopnea Index [AHI]) and sleep-related hypoxemia (percentage of total sleep time at <90% oxygen saturation [TST <90]).

**Main Outcomes and Measures:**

Outcomes were SARS-CoV-2 infection and WHO-designated COVID-19 clinical outcomes (hospitalization, use of supplemental oxygen, noninvasive ventilation, mechanical ventilation or extracorporeal membrane oxygenation, and death).

**Results:**

Of 350 710 individuals tested for SARS-CoV-2, 5402 (mean [SD] age, 56.4 [14.5] years; 3005 women [55.6%]) had a prior sleep study, of whom 1935 (35.8%) tested positive for SARS-CoV-2. Of the 5402 participants, 1696 were Black (31.4%), 3259 were White (60.3%), and 822 were of other race or ethnicity (15.2%). Patients who were positive vs negative for SARS-CoV-2 had a higher AHI score (median, 16.2 events/h [IQR, 6.1-39.5 events/h] vs 13.6 events/h [IQR, 5.5-33.6 events/h]; *P* < .001) and increased TST <90 (median, 1.8% sleep time [IQR, 0.10%-12.8% sleep time] vs 1.4% sleep time [IQR, 0.10%-10.8% sleep time]; *P* = .02). After overlap propensity score–weighted logistic regression, no SDB measures were associated with SARS-CoV-2 positivity. Median TST <90 was associated with the WHO-designated COVID-19 ordinal clinical outcome scale (adjusted odds ratio, 1.39; 95% CI, 1.10-1.74; *P* = .005). Time-to-event analyses showed sleep-related hypoxia associated with a 31% higher rate of hospitalization and mortality (adjusted hazard ratio, 1.31; 95% CI, 1.08-1.57; *P* = .005).

**Conclusions and Relevance:**

In this case-control study, SDB and sleep-related hypoxia were not associated with increased SARS-CoV-2 positivity; however, once patients were infected with SARS-CoV-2, sleep-related hypoxia was an associated risk factor for detrimental COVID-19 outcomes.

## Key Points

**Question:**

Are sleep-disordered breathing and sleep-related hypoxia associated with SARS-CoV-2 infection and COVID-19 outcomes?

**Findings:**

In this case-control study of 5402 patients in a large integrated health system, sleep-disordered breathing and sleep-related hypoxia were not associated with an increased likelihood of contracting SARS-CoV-2. After accounting for confounding factors including cardiopulmonary disease, cancer, and smoking exposure, sleep-related hypoxia indices were associated with more severe COVID-19 clinical outcomes, including hospitalization and mortality, in time-to-event analyses.

**Meaning:**

These results suggest that baseline sleep-related hypoxia may portend worse clinical prognosis in COVID-19.

## Teaser

This case-control study investigates the association of sleep-disordered breathing and sleep-related hypoxia with SARS-CoV-2 infection and COVID-19 outcomes.

## Introduction

The COVID-19 pandemic continues to threaten billions of people worldwide. Since the first case of COVID-19 infection was reported in the US, different mechanistic pathways have been purported for this highly variable disease, ranging from minimally symptomatic to severe respiratory failure and death. Although the reason for this variability remains unclear, several prognostic factors for COVID-19 infection and related morbidity have been described. Chronic comorbidities, such as diabetes, obesity, hypertension, and increasing age, are associated with greater risk for COVID-19 infection.^1,2^ Likewise, hypoxemia, a pivotal pathophysiological consequence of COVID-19 pneumonitis,^3^ is the cardinal reason for hospital admissions in COVID-19 disease, with mortality rates ranging from 40% to 80% among those admitted to the intensive care unit.^4,5^

There is strong overlap between sleep-disordered breathing (SDB), afflicting an estimated 1 billion persons worldwide,^6^ and comorbidities associated with COVID-19 severity; therefore, it is not unanticipated that observational studies have associated SDB with the risk for SARS-CoV-2 viral infection and adverse COVID-19 outcomes, such as hospital admission, mechanical ventilation, and mortality.^7,8,9,10,11^ Nonetheless, when adjusted for body mass index (BMI) and comorbidities, the association in many studies was attenuated, highlighting the importance of better understanding the biological pathways of SDB and its interplay in COVID-19 disease.^12^ Moreover, SDB was often identified via diagnosis code in these studies or characterized by limited granularity of reported direct objective measures of SDB and hypoxemia severity.

It is generally recognized that the event-based Apnea-Hypopnea Index (AHI), the most commonly used parameter for defining SDB, may not sufficiently reflect the complex pathophysiological mechanisms underlying SDB or isolated influences of sleep-related hypoxia, which could explain the inconsistent findings of recent studies. Observational studies have demonstrated that measures of nocturnal hypoxemia, such as the percentage of total sleep time with oxygen saturation (Sao_2_) below 90% (TST <90), better estimates outcomes in SDB, including mortality, than the AHI.^13,14,15^ Furthermore, it is recognized that hypoxia potentiates inflammation^8,16^ and coagulation,^17^ triggers proinflammatory cytokines,^18^ and promotes viral replication.^19^ Recent findings also suggest that hypoxemia is associated with elevated inflammatory markers, ie, higher white blood cell counts, neutrophil counts, D-dimer levels, and C-reactive protein (CRP) levels, in COVID-19 disease.^20^ Therefore, it has been postulated that progressive hypoxia may act as an amplifier of COVID-19 disease.^3^

We therefore examined baseline polysomnographic measures of SDB and sleep-related hypoxia as facilitative precursors of acquiring SARS-CoV-2 infection and investigated their associations with COVID-19 adverse outcomes. Importantly, data generated may inform risk stratification strategies to mitigate COVID-19 morbidity and mortality. We leveraged our large polysomnographically phenotyped cohort to investigate (1) the association of SDB and sleep-related hypoxia with SARS-CoV-2 infection in a case-control design and (2) the association of SDB and sleep-related hypoxia with World Health Organization (WHO)-designated COVID-19^21^ clinical outcomes and times to outcome events in a retrospective cohort. We postulate that such associations persist after accounting for potential confounding influences, including obesity, underlying cardiopulmonary disease, cancer, and smoking.

## Methods

### Study Design

For the case-control study design, we first assessed the association of SDB and sleep-related hypoxia with SARS-CoV-2 positivity. Second, a retrospective cohort design was used to assess the association of SDB and sleep-related hypoxia with ordinally categorized COVID-19 clinical outcomes and times to relevant clinical events. We used an institutional review board–approved registry of patients from the Cleveland Clinic Health System (Ohio and Florida) who were tested for COVID-19 between March 8 and November 30, 2020, with available sleep study records within the Cleveland Clinic Sleep Study Registry. The exposure of interest included polysomnographic indices of SDB and hypoxia recorded from the Cleveland Clinic Sleep Study Registry, including the frequencies of apneas and hypopneas (AHI, central apnea index, and obstructive apnea index) and sleep-related hypoxemia (TST <90), mean Sao_2_, and nadir Sao_2_. This study followed the Strengthening the Reporting of Observational Studies in Epidemiology (STROBE) reporting guideline.

### Study Population

For the case-control design, we included all adult patients (≥18 years old) who tested positive by reverse transcription–polymerase chain reaction for SARS-CoV-2 at all Cleveland Clinic locations in Ohio and Florida during the study period and had a sleep study record available. Data on race and ethnicity were extracted from the electronic health record; use of race and ethnicity information was not dictated by any funding agency. For each patient with a positive SARS-CoV-2 test result, the matching algorithm identified 3 patients with a negative SARS-CoV-2 test result of the same sex, race, and ethnic group, who tested within the same testing interval, and who were within 8 years of age. For the retrospective cohort, we included all adult patients (aged ≥18 years) who had a positive test result by reverse transcription–polymerase chain reaction for SARS-CoV-2 at all Cleveland Clinic locations in Ohio and Florida during the same study period and who had a sleep study record available. A detailed description of the Cleveland Clinic COVID-19 Registry and testing protocol has been published previously (eMethods in the Supplement).^22,23^ All patients, regardless of age, who were tested for SARS-CoV-2 at all Cleveland Clinic locations in Ohio and Florida (which includes >220 outpatient locations and 18 hospitals in Ohio and Florida) were included in the registry. A waiver of informed consent from study participants in the COVID-19 registry was granted by the Cleveland Clinic institutional review board. The waiver was granted as these analyses were based on data already collected in the COVID-19 registry.

### Data Collection

#### Sleep Testing and Registry

Attended in-laboratory polysomnography, split night polysomnography (diagnostic portion considered), or unattended type III portable sleep studies were conducted in accordance with the American Academy of Sleep Medicine guidelines^24^ using Polysmith software (Nihon Kodhen). Respiratory events were defined based on the American Academy of Sleep Medicine scoring guidelines (eMethods in the Supplement).^24^

The AHI was determined by the frequency of these respiratory events per hour of the total diagnostic sleep time during polysomnography. The primary sleep-related hypoxia measure was TST <90, given its association with poor outcomes in previous epidemiological studies.^3,19,25^ The value of TST <90 was divided by median and quartiles owing to the skewed nature of this variable. Other hypoxia measures examined include mean Sao_2_, Sao_2_ nadir, wake supine end-tidal carbon dioxide (ETCO_2_), and time spent with an ETCO_2_ level greater than or equal to 50 mm Hg, along with the minimum and maximum values. For type III portable sleep studies, recording time instead of sleep time was used. Natural language processing^26^ was used to obtain documentation of continuous positive airway pressure (PAP) usage at the time of the first SARS-CoV-2 test.

#### Study Outcomes

Our main outcomes were SARS-CoV-2 infection and WHO-designated COVID-19 clinical outcomes (hospitalization, use of supplemental oxygen, noninvasive ventilation, mechanical ventilation or extracorporeal membrane oxygenation, and death). We also performed a secondary analysis to investigate the association of SDB and sleep-related hypoxia with the composite event of hospitalization or death.

### Statistical Analysis

Data are presented as mean (SD) or median (IQR) for continuous variables and counts (percentages) for categorical variables. Comparisons of demographic details, comorbidities, clinical characteristics, and SDB indices between the 2 groups (AHI <15 and AHI ≥15 events/h) were made using a 2-sample *t* test or Wilcoxon rank-sum test based on distribution for continuous variables, whereas a Pearson χ^2^ test or Fisher exact test was used for categorical variables. Given the skewed nature of TST <90, this measure was categorized by median and across quartiles.

First, a propensity score for SARS-CoV-2 positivity was estimated from a multivariable logistic regression model including patient age, sex, race, BMI (calculated as weight in kilograms divided by height in meters squared), diabetes, hypertension, coronary artery disease, heart failure, asthma, chronic obstructive pulmonary disease, cancer, smoking (pack-years), and duration from sleep study to COVID-19 test. As patients with SDB are more likely to have obesity and have more comorbidities, we performed overlap propensity score weighting to account for confounding influences between patients who tested positive vs negative for COVID-19.^27^ Overlap propensity score–weighted logistic regression models were used to investigate associations between testing positive for SARS-CoV-2 and sleep indices.

Second, ordinal logistic regression was used to assess the association between the maximum observed WHO-designated clinical outcomes COVID-19 score on an ordinal scale (1 = not hospitalized, no limitation of activities [or resumption of normal activity]; 2 = not hospitalized but limitation on activities; 3 = hospitalized, not requiring supplemental oxygen; 4 = hospitalized, requiring supplemental oxygen [low flow, eg, nasal prong]; 5 = hospitalized, requiring noninvasive ventilation and/or high-flow oxygen; 6 = hospitalized, on invasive ventilation or extracorporeal membrane oxygenation; and 7 = death), modified from WHO-designated clinical outcomes^21^ with SDB indices. Both unadjusted and multivariable models were examined. Proportional odds assumption was checked for all models.

Third, because SDB and sleep-related hypoxic chronic exposures may accelerate worsening of clinical outcomes after contracting SARS-CoV-2, and as a way to attempt to understand how SDB and sleep-related hypoxia influence COVID-19 clinical outcomes, time-to-event analysis was performed for the composite event of hospitalization or death using Kaplan-Meier approaches, and comparison between groups was performed using the log-rank test. The start time was date of symptom onset. If no symptom-onset information was available, COVID-19 testing date was used as the start point. Patients lost to follow-up were assumed to have no hospitalization or death event within 90 days after COVID-19 testing. A Cox proportional hazards model was used to evaluate the association between hospitalization or death and sleep indices, with and without adjustment of covariates, after testing of the proportional hazards assumption.

Secondary analyses were conducted to examine interactions of demographic factors, in addition to sensitivity analyses to take into consideration PAP usage, hypopnea rule, type III sleep apnea testing, timing of sleep study, losses to follow-up, and stratified analyses (eMethods in the Supplement). All statistical analyses were performed based on an overall significance level of .05, and tests were 2-sided. Analyses were conducted using SAS software, version 9.4 (SAS Institute Inc).

## Results

**Figure 1.**
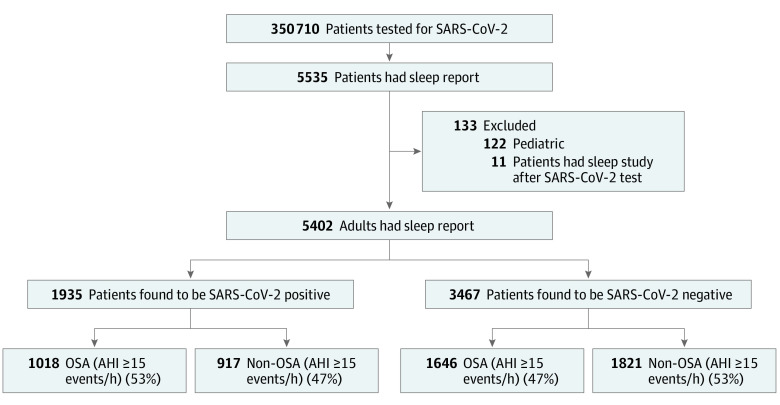
Flowchart of Patients Positive and Negative for SARS-CoV-2 AHI indicates Apnea-Hypopnea Index; OSA, obstructive sleep apnea.

Of a total of 350 710 individuals who were tested for SARS-CoV-2, 5402 had an available sleep study, including 1935 (35.8%) who tested positive for SARS-CoV-2. Among those who tested positive, 1018 (52.6%) had SDB (AHI ≥15 events/h) (Figure 1; eTables 1 and 2 in the Supplement). Demographic and clinical characteristics of patients who tested positive and negative for SARS-CoV-2 are shown in Table 1. For the 5402 included participants, the mean (SD) age was 56.4 (14.5) years; 3005 (55.6%) were women and 2397 (44.4%) were men; and 1696 were Black (31.4%), 3259 were White (60.3%), and 822 were of other race or ethnicity (15.2%), which included American Indian or Alaska Native, Asian, Native Hawaiian or Other Pacific Islander, and multiracial. Patients who tested positive for SARS-CoV-2 were less likely to be women (980 of 1935 [50.6%] vs 2025 of 3467 [58.4%]; *P* < .001) but were more likely to be White (1236 of 1935 [63.9%] vs 2023 of 3467 [58.4%]; *P* < .001) and have a higher BMI (mean [SD] BMI, 35.9 [9.2] vs 34.7 [9.0]; *P* < .001) than those who tested negative for SARS-CoV-2. More detailed results of the cohorts and additional secondary analyses can be found in the eResults in the Supplement.

**Table 1.** Comparison of Characteristics in Patients Who Tested Positive and Negative for SARS-CoV-2

Factor	Negative (n = 3467)		Positive (n = 1935)		*P* value
	Total No.	Statistics	Total No.	Statistics	
Age, mean (SD), y	3467	56.5 (15.3)	1935	56.4 (14.5)	.81^a^
Female sex, No. (%)	3467	2025 (58.4)	1935	980 (50.6)	<.001^b^
Male sex, No. (%)		1442 (41.6)		955 (49.4)	
Race, No. (%)					
Black or African American	3467	1159 (33.4)	1935	537 (27.8)	<.001^b^
White		2023 (58.4)		1236 (63.9)	
Other^c^		285 (8.2)		162 (8.4)	
Health care system, No. (%)					
Ohio	3467	3281 (94.6)	1935	1870 (96.6)	<.001^b^
Florida		186 (5.4)		65 (3.4)	
BMI, mean (SD)	3451	34.7 (9.0)	1919	35.9 (9.2)	<.001^d^
Comorbidities, No. (%)^b^					
Coronary artery disease	3467	791 (22.8)	1935	335 (17.3)	<.001
Hypertension		2431 (70.1)		1157 (59.8)	
Heart failure		667 (19.2)		256 (13.2)	
Asthma		1243 (35.9)		506 (26.1)	
COPD/emphysema		618 (17.8)		275 (14.2)	
Cancer		771 (22.2)		313 (16.2)	
Diabetes		1278 (36.9)		613 (31.7)	
Smoking, No. (%)					
No	3467	1707 (49.2)	1935	1265 (65.4)	<.001^b^
Current smoker		409 (11.8)		100 (5.2)	
Former smoker		1351 (39.0)		570 (29.5)	
Smoking pack-years, No. (%)					
Never	3467	2214 (63.9)	1935	1520 (78.6)	<.001^e^
0 to <10		423 (12.2)		140 (7.2)	
10 to <30		485 (14.0)		152 (7.9)	
≥30		345 (10.0)		123 (6.4)	
Epworth Sleepiness Scale score, mean (SD)	3255	9.9 (5.4)	1789	9.8 (5.3)	.68^d^
Duration sleep study before COVID test, median (IQR), y	3467	4.0 (1.9-7.0)	1935	4.7 (2.5-7.9)	<.001^e^
Sleep procedure type, No. (%)					
PSG	3236	1074 (33.2)	1782	520 (29.2)	.01^b^
Split		1833 (56.6)		1066 (59.8)	
Type III		329 (10.2)		196 (11.0)	
PAP use on COVID test, No. (%)	3467	537 (15.5)	1935	325 (16.8)	.21^b^
TST, mean (SD), min	3405	328.2 (90.5)	1894	331.9 (88.5)	.15^d^
AHI, median (IQR), event/h	3467	13.6 (5.5-33.6)	1935	16.2 (6.1-39.5)	<.001^e^
AHI ≥15 event/h, No. (%)	3467	1646 (47.5)	1935	1018 (52.6)	<.001^b^
AHI categories, No. (%)					
0 to <5	3467	700 (20.2)	1935	345 (17.8)	<.001^e^
5 to <15		1121 (32.3)		572 (29.6)	
15 to <30		683 (19.7)		383 (19.8)	
≥30		963 (27.8)		635 (32.8)	
AHI, median (IQR), event/h					
Central	2068	0.00 (0.00-0.20)	1068	0.00 (0.00-0.20)	.22^e^
Obstructive	2783	0.48 (0.00-2.4)	1556	0.60 (0.00-3.1)	.01^e^
TST <90					
Median (IQR), %	3176	1.4 (0.10-10.8)	1853	1.8 (0.10-12.8)	.02^e^
>1.5%, No. (%)	3176	1551 (48.8)	1853	956 (51.6)	.06^b^
Quartiles, No. (%)					
0-0.1	3176	945 (29.8)	1853	483 (26.1)	.01^e^
0.2-1.5		680 (21.4)		414 (22.3)	
1.6-11.8		785 (24.7)		468 (25.3)	
11.9-100		766 (24.1)		488 (26.3)	
Sao_2_, mean (SD), %					
Mean	3283	93.0 (3.4)	1811	93.1 (2.7)	.50^a^
Minimum	3344	83.5 (8.0)	1851	83.1 (7.9)	.03^d^
Maximum ETCO_2_ during sleep, mean (SD)	1021	48.9 (14.7)	527	48.8 (6.9)	.85^a^

Abbreviations: AHI, Apnea-Hypopnea Index; BMI, body mass index (calculated as weight in kilograms divided by height in meters squared); COPD, chronic obstructive pulmonary disease; ETCO_2_, end-tidal carbon dioxide; PAP, positive airway pressure; PSG, polysomnography pressure; Sao_2_, oxygen saturation; TST, total sleep time; TST <90, total sleep time spent with Sao_2_<90%.

^a^ Determined by Satterthwaite *t* test.

^b^ Determined by Pearson χ^2^ test.

^c^ Other includes American Indian or Alaska Native, Asian, Native Hawaiian or other Pacific Islander, and multiracial.

^d^ Determined by *t* test.

^e^ Determined by Wilcoxon rank-sum test.

### Sleep Apnea Measures and SARS-CoV-2 Positivity

To investigate the association between sleep apnea measures and SARS-CoV-2 positivity, we compared positive SARS-CoV-2 cases to controls (negative for SARS-CoV-2) in multivariable logistic regression models. Patients who tested positive vs negative for SARS-CoV-2 were more likely to have SDB (AHI ≥15 events/h; 52.6% vs 47.5%; *P* = .001), higher AHI (median events/h, 16.2 [IQR, 6.1-39.5] vs 13.6 [IQR, 5.5-33.6]; *P* < .001), an increased TST <90 (median, 1.8% sleep time [IQR, 0.10%-12.8% sleep time] vs 1.4% sleep time [IQR, 0.10%-10.8% sleep time]; *P* = .02), and a lower minimum Sao_2_ (mean [SD], 83.1% [7.9%] vs 83.5% [8.0%]; *P* = .03). When TST <90 was categorized by quartiles, increasing TST <90 was associated with increased SARS-CoV-2 positivity. However, there was no significant difference in mean Sao_2_, maximum ETCO_2_ during sleep, and PAP usage by SARS-CoV-2 test status (Table 1). After overlap propensity score–weighted logistic regression, none of the sleep apnea measures were associated with SARS-CoV-2 positivity (eTable 3 in the Supplement).

### Association of Sleep Apnea Measures With COVID-19 Clinical Outcomes

In ordinal logistic regression analysis, SDB—defined by an AHI greater than or equal to 15 events per hour—was significantly associated with worse WHO-designated clinical outcomes COVID-19 ordinal scale scores in the univariable model. However, in the multivariable fully adjusted model, this association was no longer statistically significant (odds ratio [OR], 1.00; 95% CI, 0.80-1.25; *P* = .99) (Table 2).

**Table 2.** Ordinal Logistic Models of World Health Organization Outcome (N = 1935)

Independent variable	Model 1: univariable		Model 2: multivariable after adjustment of age, sex, race, and BMI		Model 3: multivariable after adjustment of age, sex, race, BMI, comorbidities, and health care system site^a^	
	OR (95% CI)	*P* value	OR (95% CI)	*P* value	OR (95% CI)	*P* value
Sleep-disordered breathing frequency measure (AHI)						
AHI, 5 event/h increment	1.02 (1.01-1.04)	.006	1.00 (0.98-1.02)	.93	0.99 (0.97-1.01)	.53
AHI ≥15 vs <15	1.40 (1.15-1.70)	<.001	1.03 (0.83-1.29)	.79	1.00 (0.80-1.25)	.99
AHI categories, event/h						
5-10 vs 0-5	1.67 (1.21-2.30)	.002	1.27 (0.90-1.79)	.17	1.21 (0.8-1.72)	.29
15-30 vs 0-5	1.89 (1.35-2.66)	<.001	1.30 (0.90-1.88)	.16	1.28 (0.8-1.87)	.19
>30 vs 0-5	2.01 (1.47-2.74)	<.001	1.17 (0.82-1.68)	.39	1.06 (0.73-1.53)	.76
Sleep-related hypoxia measures						
TST <90, median >1.8% vs ≤1.8%, % sleep time	1.95 (1.59-2.39)	<.001	1.55 (1.24-1.93)	<.001	1.39 (1.10-1.74)	.005
TST <90, quartiles						
0.2-1.8 vs 0-0.1	1.61 (1.18-2.20)	.003	1.23 (0.89-1.72)	.21	1.21 (0.86-1.69)	.27
1.9-12.8 vs 0-0.1	2.38 (1.77-3.21)	<.001	1.59 (1.15-2.21)	.005	1.54 (1.10-2.14)	.01
12.9-100 vs 0-0.1	2.62 (1.95-3.52)	<.001	1.93 (1.39-2.67)	<.001	1.55 (1.112.18)	.01
Mean Sao_2_, 5% increment	0.60 (0.50-0.71)	<.001	0.65 (0.53-0.79)	<.001	0.73 (0.60-0.90)	.003
Sao_2_ nadir, 5% increment	0.86 (0.81-0.91)	<.001	0.91 (0.85-0 .98)	.008	0.93 (0.87-1.00)	.04
TST <90, 5% increment	1.05 (1.03-1.08)	<.001	1.04 (1.01-1.07)	.002	1.02 (1.00-1.05)	.11

Abbreviations: AHI, Apnea-Hypopnea Index; BMI, body mass index; OR, odds ratio; Sao_2_, oxygen saturation; TST <90, total sleep time <90% oxygen saturation.

^a^ Comorbidities: diabetes, hypertension, coronary artery disease, heart failure, cancer, asthma, chronic obstructive pulmonary disease/emphysema, and smoking pack-years.

Sleep-related hypoxia measures were significantly associated with increasing WHO-designated clinical outcomes COVID-19 ordinal scale scores even after adjusting for patient characteristics, BMI, comorbidities, smoking history, and health care system site (Table 2). Specifically, in adjusted analyses, median TST <90 was significantly associated with increasing WHO-designated clinical outcomes COVID-19 ordinal scale scores (ie, worse outcomes, OR, 1.39; 95% CI, 1.10-1.74; *P* = .005). Patients with TST <90 between 1.8% and 12.8% and greater than 12.8% had 54% and 55% greater odds, respectively, of a higher level in the WHO-designated clinical outcomes COVID-19 ordinal scale than those with TST <90 of less than or equal to 0.1% (OR, 1.54; 95% CI, 1.10-2.14; *P* = .011 and OR, 1.55; 95% CI, 1.11-2.18; *P* = .01, respectively). When the mean Sao_2_ increased by 5%, the odds of the WHO-designated clinical outcomes COVID-19 ordinal scale score decreased 27% in the fully adjusted model (OR, 0.73; 95% CI, 0.60-0.90; *P* = .003).

### Sleep Apnea Measures and COVID-19 Hospitalizations and Mortality

**Figure 2.**
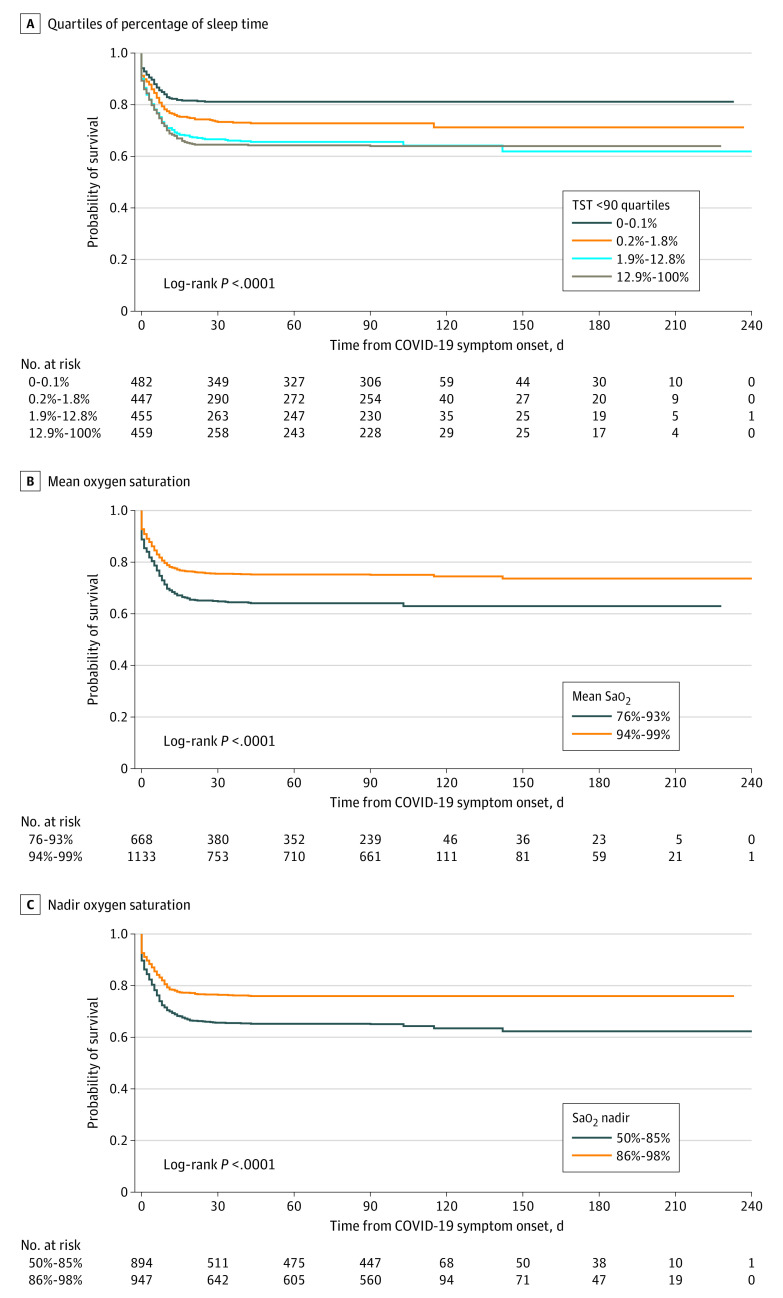
Kaplan-Meier Curves of Sleep-Related Hypoxia Associated With COVID-19 Hospitalization and Mortality Unadjusted Kaplan-Meier curves are presented. A, Quartiles of percentage of sleep time at <90% oxygen saturation (Sao_2_); B, mean Sao_2_; C, nadir Sao_2_. TST <90 indicates total sleep time spent with Sao_2_ at <90%.

Using the log-rank test, we observed increased rates of hospitalization and death from the time of COVID-19 symptom onset in patients with baseline sleep-related hypoxia (Figure 2). Cox proportional hazards models of hospitalization and death for different apnea measures are shown in Table 3. Sleep-related hypoxia, defined by a TST <90 level of greater than 1.8%, was associated with a 31% higher risk of hospitalization and mortality than a TST <90 level of less than or equal to 1.8% (hazard ratio [HR], 1.31; 95% CI, 1.08-1.57; *P* = .005). Patients with TST <90 between 1.8% and 12.8% and greater than 12.8% had a 42% and 38% higher risk of hospitalization and mortality, respectively, than those with a TST <90 of less than or equal to 0.1% (HR, 1.42; 95% CI, 1.08-1.87; *P* = .013 and HR, 1.38; 95% CI, 1.05-1.83; *P* = .02, respectively). Similarly, there was a 19% and 6% decrease in risk of hospitalization and mortality for each 5% increment in mean and nadir Sao_2_ (HR, 0.81; 95% CI, 0.69-0.94; *P* = .007 and HR, 0.94; 95% CI, 0.89-1.00; *P* = .04, respectively).

**Table 3.** Cox Proportional Hazard Models of Hospitalization and Death (N = 1925)

Independent variable	Model 1: univariable		Model 2: multivariable after adjustment of age, sex, race, and BMI		Model 3: multivariable after adjustment of age, sex, race, BMI, comorbidities, and health care system site^a^	
	HR (95% CI)	*P* value	HR (95% CI)	*P* value	HR (95% CI)	*P* value
Sleep-disordered breathing frequency measure (AHI)						
AHI, 5 event/h increment	1.02 (1.01-1.03)	.006	1.01 (0.99-1.02)	.43	1.00 (0.98-1.02)	.94
AHI ≥15 vs <15	1.30 (1.10-1.54)	.003	1.04 (0.87-1.25)	.67	1.01 (0.84-1.21)	.95
AHI categories, event/h						
5-10 vs 0-5	1.51 (1.14-2.01)	.004	1.18 (0.88-1.57)	.26	1.14 (0.85-1.53)	.37
15-30 vs 0-5	1.62 (1.20-2.20)	.002	1.15 (0.84-1.56)	.38	1.12 (0.82-1.53)	.48
>30 vs 0-5	1.76 (1.33-2.32)	<.001	1.19 (0.88-1.59)	.25	1.10 (0.81-1.49)	.53
Sleep-related hypoxia measures						
TST <90 by median >1.8% vs ≤1.8%, % sleep time	1.67 (1.40-1.99)	<.001	1.39 (1.16-1.68)	<.001	1.31 (1.08-1.57)	.005
TST <90 quartiles						
0.1-1.8 vs 0-0.1	1.51 (1.15-2.00)	.003	1.12 (0.84-1.48)	.44	1.13 (0.85-1.50)	.40
1.9-12.8 vs 0-0.1	2.03 (1.56-2.64)	<.001	1.40 (1.07-1.84)	.015	1.42 (1.08-1.87)	.01
12.9-100 vs 0-0.1	2.11 (1.63-2.74)	<.001	1.59 (1.21-2.09)	<.001	1.38(1.05-1.83)	.02
Mean Sao_2_, 5% increment	0.69 (0.60-0.80)	<.001	0.73 (0.63-0.85)	<.001	0.81 (0.69-0.94)	.007
Sao_2_ nadir, 5% increment	0.89 (0.85-0.94)	<.001	0.93 (0.88-0.99)	.01	0.94 (0.89-1.00)	.04
TST <90, 5% increment	1.04 (1.02-1.06)	<.001	1.03 (1.01-1.05)	.001	1.02 (1.00-1.04)	.06

Abbreviations: AHI, Apnea-Hypopnea Index; BMI, body mass index; HR, hazard ratio; Sao_2_, oxygen saturation; TST <90, total sleep time <90% oxygen saturation.

^a^ Comorbidities: diabetes, hypertension, coronary artery disease, heart failure, cancer, asthma, chronic obstructive pulmonary disease/emphysema, and smoking pack-years.

### Secondary Analysis

We observed a significant association between mean Sao_2_ and sex after adjustment of subject characteristics, comorbidities, and health care system site. Women had a 46% decreased odds of a higher WHO-designated clinical outcomes COVID-19 ordinal scale when the mean Sao_2_ increased by 5% (OR, 0.54; 95% CI, 0.40-0.73; *P* < .001). However, in men, this association was attenuated and not statistically significant (OR, 0.88; 95% CI, 0.67-1.14; *P* = .33). There was no statistically significant association in the time-to-event analysis between other sleep variables and age, sex, race, and BMI in adjusted models.

After exclusion of those individuals receiving PAP therapy, associations between sleep apnea measures, COVID-19 clinical outcomes, hospitalizations, and mortality were consistent with the primary results (eTables 4 and 5 in the Supplement). Similar findings were also found when restricting analyses to patients with sleep studies using the 3% hypopnea scoring rule (eTables 6 and 7 in the Supplement) and in those undergoing in-laboratory sleep testing, as it is known that type III sleep studies can underestimate the severity of SDB compared with polysomnography (eTables 8 and 9 in the Supplement).^28^ Similar results were found in those with a sleep study performed within 5 years of the COVID-19 test vs more than 5 years earlier (eTables 10, 11, 12, and 13 in the Supplement). Additionally, we found that mean Sao_2_ and minimum Sao_2_ had significant natural indirect association with CRP, suggesting that CRP may represent a mediator in the association of high-level WHO-designated clinical outcomes COVID-19 classification with sleep-related hypoxia (eTable 14 in the Supplement). Overall, obstructive sleep apnea and central sleep apnea subtype analyses were consistent with the findings of the models that defined SDB using AHI, and no significant findings were observed with ETCO_2_ values and outcomes (eTables 15 and 16 in the Supplement).

Clinical characteristics of patients who were lost to follow-up are described in eTable 17 in the Supplement. After using multiple imputation to address missing variables and excluding patients who were lost to follow-up, results were consistent with primary results, ie, that hypoxia measures (TST <90 by median, TST <90 quartiles between 1.8% and 12.8% and >12.8% vs TST <90 of ≤0.1%, and mean Sao_2_ increased by 5%) were associated with higher-level WHO-designated clinical outcomes COVID-19 ordinal scale classification and increased risk for hospitalization and death (eTables 18, 19, and 20 in the Supplement). Asymptomatic patients had a slightly stronger association between sleep-related hypoxia measures and higher WHO-designated clinical outcomes COVID-19 ordinal scale classification and increased risk for hospitalization and death in a stratified analysis (eTables 21 and 22 in the Supplement).

## Discussion

In this cohort of patients from a large integrated health system, sleep-related hypoxemia was identified as a risk factor for increased severity of COVID-19 clinical outcomes, including hospitalization and mortality. The traditional frequency measure of SDB, ie, AHI, was associated with SARS-CoV-2 positivity in logistic regression models; however, when using the overlap weighting propensity-matched approach, SDB, defined by AHI and hypoxemia measures, was not significantly associated with SARS-CoV-2 positivity. Both ordinal logistic regression and time-to-event analyses identified sleep-related hypoxemia as consistently associated with increased severity of COVID-19 outcomes even after adjustment for confounding influences, including cardiopulmonary disease and smoking history, the latter identified to represent a risk for poor COVID-19 outcomes.^16^

Our findings suggest that baseline sleep-related hypoxia is associated with progression of hypoxic insult and hypoxia-related injury in COVID-19 pathophysiology, hence serving as an amplifier.^20^ Although hypoxia potentiates both viral replication^29,30^ and inflammation,^15^ our findings suggest that baseline sleep-related hypoxia is not associated with increased likelihood of contracting SARS-CoV-2; however, it may play a role in worse outcomes once the viral illness evolves. Hypoxia contributors in COVID-19 are likely multifactorial, including microinfarcts, pulmonary parenchymal inflammation, hypoxic pulmonary vasoconstriction, and lung injury,^31,32^ which may influence different treatment outcomes.^33^ Our results are consistent with the observation that hypoxemia (Sao_2_<90% despite oxygen supplementation) is associated with mortality in critically ill patients with COVID-19, particularly as we show persistence of findings after exclusion of those on PAP therapy.^34^ Lower thresholds of hypoxic exposure (ie, defined by a TST <90 of >0.5%) have been associated with adverse clinical outcomes such as cognitive impairment.^35^ When we examined the lower limit of the fourth quartile, ie, greater than 12.8%, comparable levels of hypoxic exposure have been implicated in cardiac structural abnormalities such as left ventricular hypertrophy.^36^

We found a minimal attenuation in the association of sleep-related hypoxia measures with WHO-designated clinical outcomes COVID-19 clinical outcomes when PAP therapy was excluded from the analysis; however, we believe this was due to limitations of sample size and suboptimal adherence and was potentially attributable to the lesser degree of hypoxia in non-PAP users compared with PAP users. Residual hypoxia despite treatment may provide a potential explanation for the worse COVID-19 outcomes and could be the potential reason for poor outcomes reported in patients with treated SDB.^10^ Our current results, however, suggest that chronic exposure to sleep-related hypoxia may serve as a priming mechanism to the untoward consequences of COVID-19 illness. Of note, the current findings are not consistent with the previously described potential salutatory effects of hypoxic preconditioning in COVID-19 promoting hypoxic tolerance.^30^ Also, results indicate that chronic sleep-related hypoxic exposures not only contribute to increased likelihood of COVID-19 morbidity and mortality but also suggest more rapid clinical decompensation from the time of SARS-CoV-2 testing.

As results persisted after accounting for cardiopulmonary disease and smoking history, nocturnal hypoxemia may represent a priority, clinically relevant component of sleep apnea–related physiologic stress portending worse COVID-19 clinical outcomes. Sleep-related hypoxia may more accurately capture SDB-related physiologic stress compared with the AHI which does not reflect duration and severity of hypoxic exposures. Intermittent hypoxia is implicated in sympathetic activation, endothelial dysfunction, systemic inflammation, and oxidative stress,^16,37^ ie, pathways postulated to contribute to COVID-19 morbidity and mortality. Clinical and epidemiological data identify sleep-related intermittent hypoxia and extended periods of oxygen desaturations in incident cardiovascular disease and all-cause mortality^15,29,34^ thereby lending credence to biological plausibility in accelerating negative COVID-19 consequences. Our data support CRP, a proinflammatory factor implicated in intermittent hypoxia pathways and COVID-19 pathophysiology,^38,39^ as a significant mediator of sleep-related hypoxia and COVID-19 morbidity and mortality, suggesting its role as a relevant intermediary mechanism.

### Strengths and Limitations

One of the strengths of this study was the inclusion of a large cohort across multiple hospital sites as well as the COVID-19 and sleep data registries, which allowed for greater specificity of phenotyping of SDB and hypoxia. As such, unlike in other studies,^40^ our results suggest that capturing the counts or frequency of respiratory events irrespective of obstructive or central origin may have been less useful and informative than degree of hypoxia in estimating adverse COVID-19 clinical outcomes.

This study had several limitations. Owing to the retrospective nature of this study, it was susceptible to referral and selection biases and unmeasured confounding (ie, including those persons who presented clinically with a sleep disorder, which warranted sleep and SARS-CoV-2 testing); thus, findings are not generalizable for that reason. Another limitation was the consideration of a single night of sleep testing rather than multiple nights of sleep testing to ascertain sleep-exposure measures, which may have limited the ability to accurately ascertain night-to-night variability of SDB physiology.^41^ Similarly, inclusion of both in-laboratory and portable sleep testing, use of PAP therapy, and use of both greater than 3% and 4% hypopnea rules are limitations. We addressed these limitations by conducting sensitivity analyses, which showed results consistent with primary findings. We were unable to adjust for COVID-19 medication use given the close linkage with the outcomes. Additionally, there may have been residual confounding, as diagnosis of cardiopulmonary disease and smoking pack-year history is based on the electronic medical record data extraction and may carry some inaccuracies despite the validated approaches of natural language processing that were used.^42^

## Conclusions

Results of this case-control study revealed that SDB and sleep-related hypoxia were not associated with increased SARS-CoV-2 positivity; however, once patients were infected with SARS-CoV-2, sleep-related hypoxia was an associated risk factor for detrimental COVID-19 outcomes. Strategies to better discern sleep apnea-specific hypoxic stress are needed, such as use of sleep apnea–related hypoxic burden as a more sensitive biomarker of sleep apnea–related hypoxia, as the latter is recognized to be associated with increased cardiovascular mortality.^14^ Furthermore, understanding how sleep-related hypoxia may influence vaccine efficacy as well as elucidating underlying hypoxic mechanisms portending more severe COVID-19 disease and mortality are salient future directions. The current findings set the stage for interventional studies to identify whether early, effective PAP or supplemental oxygen administration in those with high nocturnal hypoxic physiological stress improves COVID-19 outcomes. If sleep-related hypoxia indeed translates to worse COVID-19 outcomes, risk stratification strategies should be implemented to prioritize early allocation of COVID-19 therapy to this subgroup of patients.

## Notes

Supplement.
eMethods.

eResults.
**eTable 1. **Patient Characteristics of SARS-CoV-2 Positive Patients **eTable 2. **Data Summary by WHO-7 Ordinal Scale Clinical Outcome Level **eTable 3. **Logistic Models for Association Between SARS-CoV-2 Test Outcome and Sleep Apnea Measures Using Propensity Score Overlap Weighting **eTable 4. **Ordinal Logistic Models of WHO-7 Outcome Before and After Adjustment, for Adult Patients Without PAP Use on COVID-19 Testing **eTable 5. **Cox Proportional Hazard Models of Hospitalization and Death Without PAP Use on COVID-19 Testing **eTable 6. **Ordinal Logistic Models of WHO-7 Outcome Using the 3% Hypopnea Rule **eTable 7. **Cox Proportional Hazard Models of Hospitalization and Death Using 3% Hypopnea Rule **eTable 8.** Ordinal Logistic Models of WHO-7 Outcome on Patients Having Polysomnogram **eTable 9.** Cox Proportional Hazard Models of Hospitalization and Death on Patients Having Polysomnogram **eTable 10.** Ordinal Logistic Models of WHO-7 Outcome on Patients Who Had a Sleep Study Within 5 Years of the COVID-19 Test **eTable 11.** Cox Proportional Hazard Models of Hospitalization and Death on Patients Who Had a Sleep Study Within 5 Years of the COVID-19 Test **eTable 12.** Ordinal Logistic Models of WHO-7 Outcome on Patients Who Had a Sleep Study More Than 5 Years of the COVID-19 Test **eTable 13.** Cox Proportional Hazard Models of Hospitalization and Death on Patients Who Had a Sleep Study More Than 5 Years of the COVID-19 Test **eTable 14.** Logistic Models of Any High-Level WHO Scale **eTable 15.** Ordinal Logistic Models of WHO-7 Outcome **eTable 16.** Cox Proportional Hazard Models of Hospitalization and Death **eTable 17.** Comparison by Lost to Follow-Up vs Censoring With Follow-Up in Time-to-Event Analysis of Hospitalization/Death **eTable 18.** Ordinal Logistic Models of WHO Outcome Before and After Adjustment – After Multiple Imputation **eTable 19.** Cox Proportional Hazard Models of Hospitalization/Death Before and After Adjustment – After Multiple Imputation **eTable 20.** Cox Proportional Hazard Models of Hospitalization/Death Before and After Adjustment **eTable 21.** Ordinal Logistic Models of WHO-7 Outcome **eTable 22.** Cox Proportional Hazard Models of Hospitalization and Death
eReferences
