# Supplementary material for: Association of Sleep-Related Hypoxia With Risk of COVID-19 Hospitalizations and Mortality in a Large Integrated Health System
Source: JAMA Netw Open. 2021 Nov 10;4(11):e2134241. doi: 10.1001/jamanetworkopen.2021.34241 (PMC8581726; doi:10.1001/jamanetworkopen.2021.34241)
Supplement: Supplement. — eMethods. eResults. eTable 1. Patient Characteristics of SARS-CoV-2 Positive Patients eTable 2. Data Summary by WHO-7 Ordinal Scale Clinical Outcome Level eTable 3. Logistic Models for Association Between SARS-CoV-2 Test Outcome and Sleep Apnea Measures Using Propensity Score Overlap Weighting eTable 4. Ordinal Logistic Models of WHO-7 Outcome Before and After Adjustment, for Adult Patients Without PAP Use on COVID-19 Testing eTable 5. Cox Proportional Hazard Models of Hospitalization and Death Without PAP Use on COVID-19 Testing eTable 6. Ordinal Logistic Models of WHO-7 Outcome Using the 3% Hypopnea Rule eTable 7. Cox Proportional Hazard Models of Hospitalization and Death Using 3% Hypopnea Rule eTable 8. Ordinal Logistic Models of WHO-7 Outcome on Patients Having Polysomnogram eTable 9. Cox Proportional Hazard Models of Hospitalization and Death on Patients Having Polysomnogram eTable 10. Ordinal Logistic Models of WHO-7 Outcome on Patients Who Had a Sleep Study Within 5 Years of the COVID-19 Test eTable 11. Cox Proportional Hazard Models of Hospitalization and Death on Patients Who Had a Sleep Study Within 5 Years of the COVID-19 Test eTable 12. Ordinal Logistic Models of WHO-7 Outcome on Patients Who Had a Sleep Study More Than 5 Years of the COVID-19 Test eTable 13. Cox Proportional Hazard Models of Hospitalization and Death on Patients Who Had a Sleep Study More Than 5 Years of the COVID-19 Test eTable 14. Logistic Models of Any High-Level WHO Scale eTable 15. Ordinal Logistic Models of WHO-7 Outcome eTable 16. Cox Proportional Hazard Models of Hospitalization and Death eTable 17. Comparison by Lost to Follow-Up vs Censoring With Follow-Up in Time-to-Event Analysis of Hospitalization/Death eTable 18. Ordinal Logistic Models of WHO Outcome Before and After Adjustment – After Multiple Imputation eTable 19. Cox Proportional Hazard Models of Hospitalization/Death Before and After Adjustment – After Multiple Imputation eTable 20. Cox Proportional Hazard Models of [file jamanetwopen-e2134241-s001.pdf]

Supplementary Online Content

Pena Orbea C, Wang L, Shah V, et al. Association of sleep-related hypoxia with risk of COVID-19 hospitalizations and mortality in a large integrated health system. *JAMA Netw Open*. 2021;4(11):e2134241. doi:10.1001/jamanetworkopen.2021.34241

eMethods.

eResults.

eTable 1. Patient Characteristics of SARS-CoV-2 Positive Patients

eTable 2. Data Summary by WHO-7 Ordinal Scale Clinical Outcome Level

eTable 3. Logistic Models for Association Between SARS-CoV-2 Test Outcome and Sleep Apnea Measures Using Propensity Score Overlap Weighting

eTable 4. Ordinal Logistic Models of WHO-7 Outcome Before and After Adjustment, for Adult Patients Without PAP Use on COVID-19 Testing

eTable 5. Cox Proportional Hazard Models of Hospitalization and Death Without PAP Use on COVID-19 Testing

eTable 6. Ordinal Logistic Models of WHO-7 Outcome Using the 3% Hypopnea Rule

eTable 7. Cox Proportional Hazard Models of Hospitalization and Death Using 3% Hypopnea Rule

eTable 8. Ordinal Logistic Models of WHO-7 Outcome on Patients Having Polysomnogram

eTable 9. Cox Proportional Hazard Models of Hospitalization and Death on Patients Having Polysomnogram

eTable 10. Ordinal Logistic Models of WHO-7 Outcome on Patients Who Had a Sleep Study Within 5 Years of the COVID-19 Test

eTable 11. Cox Proportional Hazard Models of Hospitalization and Death on Patients Who Had a Sleep Study Within 5 Years of the COVID-19 Test

eTable 12. Ordinal Logistic Models of WHO-7 Outcome on Patients Who Had a Sleep Study More Than 5 Years of the COVID-19 Test

eTable 13. Cox Proportional Hazard Models of Hospitalization and Death on Patients Who Had a Sleep Study More Than 5 Years of the COVID-19 Test

eTable 14. Logistic Models of Any High-Level WHO Scale

eTable 15. Ordinal Logistic Models of WHO-7 Outcome

eTable 16. Cox Proportional Hazard Models of Hospitalization and Death

eTable 17. Comparison by Lost to Follow-Up vs Censoring With Follow-Up in Time-to-Event Analysis of Hospitalization/Death

© 2021 Pena Orbea C et al. *JAMA Network Open.*

eTable 18. Ordinal Logistic Models of WHO Outcome Before and After Adjustment – After Multiple Imputation

eTable 19. Cox Proportional Hazard Models of Hospitalization/Death Before and After Adjustment – After Multiple Imputation

eTable 20. Cox Proportional Hazard Models of Hospitalization/Death Before and After Adjustment

eTable 21. Ordinal Logistic Models of WHO-7 Outcome

eTable 22. Cox Proportional Hazard Models of Hospitalization and Death

eReferences

This supplementary material has been provided by the authors to give readers additional information about their work.

Online Supplement

eMethods

Cleveland Clinic COVID-19 Registry

Demographics such as age, sex, race and clinical characteristics including body mass index kg/m^2 (BMI), comorbidities, travel history, COVID-19 exposure history, COVID-19 testing date, medications, presenting symptoms, date of symptoms onset, treatment, disease progression and outcomes were collected. Based on the available literature on COVID-19, disease variables were chosen to reflect disease characterization, progression, and proposed treatments.[1]
Data were extracted via electronic health records (EHR) through validated automated feeds[2] and manually by a study team trained on uniform sources for the study variable. The COVID-19 Research Registry team includes a "Reviewer" group and a "Quality Assurance" group. The reviewers manually abstracted and entered a subset of variables (signs and symptoms upon presentation) that cannot be automatically extracted from the electronic health record, and to verify high-priority variables such as co-morbidities that were automatically pulled into the database from the electronic health record. The Quality Assurance group provided an additional independent second tier of review. All collected data were managed by using the Research Electronic Data Capture (REDCap; Vanderbilt University, Nashville, TN) tool hosted at Cleveland Clinic.[3,4]

Laboratory Confirmation

Nasopharyngeal and oropharyngeal swab specimens were collected and pooled for testing by trained medical personnel. Infection with SARS-CoV-2 was confirmed by laboratory testing using the Centers for Disease Control and Prevention RT-PCR SARS-CoV-2 assay that was validated and had similar good performance across all the three nucleocapsid gene targets in the Cleveland Clinic Robert J. Tomsich Pathology and Laboratory Medicine Institute with correlations ranging 93.3% and 100% across various concentrations.The assay for testing used an extraction kit (MagNAPure;Roche) and 7500 Dx Real-Time PCR System instruments (Applied Biosystems). Between March 8 and 13, 2020, the tests were sent out to LabCorp in Burlington, North Carolina

Sleep Testing and Registry

The Cleveland Clinic Sleep Registry is a multimodal sleep neurocardiorespiratory biophysiologic repository containing raw and summary data of more than 200,000 sleep studies conducted in the Cleveland Clinic Sleep laboratory.

For PSG and split studies standard 10–20 electroencephalogram, electrocardiogram, electromyography of the chin and bilateral anterior tibialis muscle, electrooculography, and pulse oximetry (Nihon Kohden (PSG) Nonin 8000-SM (HSAT) with sampling rate of 25Hz) were recorded. Oral and nasal airflow were measured with a thermistor and nasal cannula. Respiratory effort was measured with plethysmography bands at the chest and abdomen, including summation channel. Unattended type III portable sleep studies involved recordings of air flow by using nasal pressure cannula, effort plethysmography abdominal and chest bands, body position using position sensors, oxygenation and heart rate using pulse oximetry.

End tidal CO2 (ETCO2) samples were obtained through oronasal cannulas (Salter Labs, Arvin CA) with side stream technology using a sampling flow of 75 ml/min with calibrated Nonin RespSense devices (Plymouth, MN) interfaced to the Polysmith system. Total system response time (including delay and rise times) was 4 seconds, and the sampling rate for the capnograph tracing was 4 Hz.

Episodes of apnea were defined as decrease in airflow by >90% for $\geq$10 seconds and further classified as obstructive, central, or mixed according to the presence or absence of breathing efforts based on the American Academy of Sleep Medicine. Episodes of hypopnea were defined as decrease in airflow by >30% for $\geq$10 seconds along with a decrease in oxygen saturation of $\geq$3% or arousals or $\geq$4% as dictated by insurer.

Overview of Secondary Analysis

© 2021 Pena Orbea C et al. *JAMA Network Open.*

In secondary analysis, we tested for interaction between sleep indices and recognized COVID-19 risks: age, sex, race and BMI. We conducted sensitivity analysis excluding data from 1) patients with use of positive airway pressure (PAP) therapy at the time of SARS-CoV-2 testing to take into consideration potential confounding more robustly by PAP usage 2) those with 4% hypopnea rule, 3) those undergoing home sleep apnea testing and 4) those who had the sleep study performed within 5 years of the COVID-19 test and 5) those who were lost to follow-up. We also performed a stratified analysis by the presence of symptoms to account for different start points between symptomatic and asymptomatic patients. To better understand obstructive and central aspects of SDB, we also examined SDB subtypes of obstructive sleep apnea (OSA) and central sleep apnea (CSA). Due to recognized association of hypoxia and up-regulation of inflammation including C-reactive protein (CRP), [5,6] mediation analysis was performed on the sub-group of patients who had blood sample of CRP on admission adjusted for demographics, comorbidities, smoking pack history and site. Finally, for missing data, multiple imputation was performed using a predictive mean matching method.

eResults

Among patients who tested positive for SARS-CoV-2, 170 (8.8%) were hospitalized not requiring oxygen, 191 (9.9%) were on supplemental oxygen, 106 (5.5%) were on high flow or noninvasive ventilation, 43 (2.2%) were on ECMO or mechanical ventilation and 43 (2.2%) died. **(e-Table 1)** Patients characteristics by WHO-7 COVID-19 ordinal scale level are summarized in **e-Table 2**.

© 2021 Pena Orbea C et al. *JAMA Network Open.*

eTable 1 shows a summary of demographics and clinical characteristics of SARS-CoV-2 patients by sleep disordered breathing severity. Nine hundred and seventeen patients (47.4%) had mild SDB (AHI<15 events/h) and 1,018 (52.6%) had moderate to severe SDB (AHI≥15 events/h).

eTable 1. Patient Characteristics of SARS-CoV-2 Positive Patients

Factor	Overall n=1,935		AHI<15 n=917		AHI≥15 n=1,018		
	N	Statistics	N	Statistics	N	Statistics	p-value
Age (yrs)	1,935	56.4 ± 14.5	917	53.5 ± 15.5	1,018	59.0 ± 13.0	*<0.001*[a2]
Gender (Male)	1,935	955 (49.4)	917	313 (34.1)	1,018	642 (63.1)	*<0.001*[c]
Race, n (%)	1,935		917		1,018		0.07[c]
White		1,236 (63.9)		566 (61.7)		670 (65.8)	
Black or African American		537 (27.8)		262 (28.6)		275 (27.0)	
Other		162 (8.4)		89 (9.7)		73 (7.2)	
Health Care System	1,935		917		1,018		*0.026*[c]
Cleveland Clinic Ohio		1,870 (96.6)		895 (97.6)		975 (95.8)	
Cleveland Clinic Florida		65 (3.4)		22 (2.4)		43 (4.2)	
Body mass index, kg/m^2	1,919	35.9 ± 9.2	907	34.3 ± 9.2	1,012	37.3 ± 9.0	*<0.001*[a1]
Comorbidities, n (%)							
Coronary Artery Disease	1,935	335 (17.3)	917	124 (13.5)	1,018	211 (20.7)	*<0.001*[c]
Hypertension	1,935	1,157 (59.8)	917	461 (50.3)	1,018	696 (68.4)	*<0.001*[c]
Heart failure	1,935	256 (13.2)	917	104 (11.3)	1,018	152 (14.9)	*0.020*[c]
Asthma	1,935	506 (26.1)	917	280 (30.5)	1,018	226 (22.2)	*<0.001*[c]
COPD/emphysema	1,935	275 (14.2)	917	137 (14.9)	1,018	138 (13.6)	0.38[c]
Cancer	1,935	313 (16.2)	917	152 (16.6)	1,018	161 (15.8)	0.65[c]
Diabetes	1,935	613 (31.7)	917	244 (26.6)	1,018	369 (36.2)	*<0.001*[c]
Smoking, n (%)	1,935		917		1,018		0.72[c]
No		1,265 (65.4)		607 (66.2)		658 (64.6)	
Yes		100 (5.2)		48 (5.2)		52 (5.1)	
Former Smoker		570 (29.5)		262 (28.6)		308 (30.3)	
Smoking pack years	1,935		917		1,018		*0.069b*
0.Never smoked		1,520 (78.6)		734 (80.0)		786 (77.2)	
0-10		140 (7.2)		74 (8.1)		66 (6.5)	
10-30		152 (7.9)		62 (6.8)		90 (8.8)	
30+		123 (6.4)		47 (5.1)		76 (7.5)	
Epworth Sleepiness Scale score	1,789	9.8 ± 5.3	825	9.7 ± 5.3	964	9.9 ± 5.3	0.40[a1]
Duration Sleep study before COVID test (years)	1,935	4.7 [2.5, 7.9]	917	4.8 [2.5, 7.7]	1,018	4.7 [2.5, 7.9]	0.89[b]
Sleep Procedure Type, n (%)	1,782		829		953		0.91[c]
PSG		520 (29.2)		241 (29.1)		279 (29.3)	
Split		1,066 (59.8)		494 (59.6)		572 (60.0)	
Type III		196 (11.0)		94 (11.3)		102 (10.7)	
Hypopnea rule	1935		917		1,018		<0.001[c]
3%		1,933 (69.0)		594 (64.8)		742 (72.9)	
4%		599 (31.0)		323 (35.2)		276 (27.1)	
PAP use on COVID test, n (%)	1,935	325 (16.8)	917	117 (12.8)	1,018	208 (20.4)	*<0.001*[c]
Total Sleep Time, min	1,894	331.9 ± 88.5	894	343.0 ± 88.3	1,000	322.0 ± 87.5	*<0.001*[a1]
Apnea Hypopnea Index	1,935	16.2 [6.1, 39.5]	917	5.8 [2.3, 9.6]	1,018	37.3 [23.3, 65.0]	*<0.001*[b]
AHI categories:	1,935		917		1,018		*<0.001*[b]
0-<5		345 (17.8)		345 (37.6)		0 (0.00)	
5-<15		572 (29.6)		572 (62.4)		0 (0.00)	
15-<30		383 (19.8)		0 (0.00)		383 (37.6)	

© 2021 Pena Orbea C et al. *JAMA Network Open.*

30+		635 (32.8)		0 (0.00)		635 (62.4)	
Central Apnea Index	1,068	0.00 [0.00, 0.20]	515	0.00 [0.00, 0.00]	553	0.00 [0.00, 0.40]	*<0.001*[b]
Obstructive Apnea Index	1,556	0.60 [0.00, 3.1]	656	0.16 [0.00, 0.64]	900	1.7 [0.31, 6.5]	*<0.001*[b]
% Sleep Time with SaO2<90% (TST<90)	1,853	1.8 [0.10, 12.8]	868	0.30 [0.00, 2.0]	985	7.0 [1.3, 24.4]	*<0.001*[b]
% Sleep Time with SaO2<90% (TST<90) dichotomized by median: > 1.8 %	1,853	921 (49.7)	868	226 (26.0)	985	695 (70.6)	*<0.001*[c]
% Sleep Time with SaO2<90% (TST<90) categories by quartiles:	1,853		868		985		*<0.001*[b]
0-0.1		483 (26.1)		383 (44.1)		100 (10.2)	
0.1-1.8		449 (24.2)		259 (29.8)		190 (19.3)	
1.8-12.8		459 (24.8)		131 (15.1)		328 (33.3)	
12.8-100		462 (24.9)		95 (10.9)		367 (37.3)	
Mean oxygen saturation, %	1,811	93.1 ± 2.7	843	94.1 ± 2.2	968	92.2 ± 2.8	*<0.001*[a2]
Minimum oxygen saturation, %	1,851	83.1 ± 7.9	870	86.8 ± 5.6	981	79.7 ± 8.2	*<0.001*[a2]
Maximum EtCO2 during sleep, mmHg	527	48.8 ± 6.9	246	48.0 ± 5.7	281	49.5 ± 7.6	*0.008*[a2]
Hospitalized, n (%)	1,929	549 (28.5)	915	232 (25.4)	1,014	317 (31.3)	*0.004*[c]
Death, n (%)	1,935		917		1,018		0.48[c]
No		735 (38.0)		356 (38.8)		379 (37.2)	
Yes		43 (2.2)		17 (1.9)		26 (2.6)	
Unknown		1,157 (59.8)		544 (59.3)		613 (60.2)	
WHO-7 COVID-19 Outcome, n (%)	1,935		917		1,018		*<0.001*[b]
1.Not Hospitalized		1,382 (71.4)		683 (74.5)		699 (68.7)	
2.Hospitalized not requiring oxygen		170 (8.8)		88 (9.6)		82 (8.1)	
3. Supplemental Oxygen		191 (9.9)		80 (8.7)		111 (10.9)	
4.High flow, Noninvasive mechanical ventilation		106 (5.5)		39 (4.3)		67 (6.6)	
5. ECMO, Invasive mechanical ventilation		43 (2.2)		10 (1.09)		33 (3.2)	
6.Death		43 (2.2)		17 (1.9)		26 (2.6)	
Remdesivir, n (%)	425	3 (0.71)	179	1 (0.56)	246	2 (0.81)	0.99[d]
Steroids, n (%)	425	338 (79.5)	179	139 (77.7)	246	199 (80.9)	0.41[c]
Hydroxychloroquine, n (%)	426	60 (14.1)	179	23 (12.8)	247	37 (15.0)	0.53[c]
Lopinavir/ritonavir (Kaletra) , n (%)	425	0 (0.00)	179	0 (0.00)	246	0 (0.00)	
Oral Ribavirin, n (%)	425	0 (0.00)	179	0 (0.00)	246	0 (0.00)	
Tocilizumab, n (%)	425	19 (4.5)	179	4 (2.2)	246	15 (6.1)	0.06[c]
C-reactive protein (CRP) on admission	466	5.3 [2.2, 10.3]	187	4.7 [1.4, 8.5]	279	5.8 [2.6, 10.8]	*0.007*[b]
Interleukin-6 on admission, pg/mL	16	12.5 [5.0, 35.1]	8	10.3 [5.0, 29.1]	8	15.0 [8.0, 38.5]	0.47[b]
Lactate dehydrogenase (LDH) on admission, units/L	345	291.0 [225.0, 393.0]	142	270.5 [210.0, 376.0]	203	311.0 [232.0, 413.0]	*0.014*[b]
Lactate on admission, mmol/L	206	1.5 [1.1, 2.0]	83	1.5 [1.1, 2.1]	123	1.5 [1.1, 1.8]	0.68[b]
Hospitalized or death	1,925	543 (28.2)	911	228 (25.0)	1,014	315 (31.1)	*0.003*[c]

Abbreviations: AHI: apnea hypopnea index; BMI: body mass index; CAI: central apnea index; OAI: obstructive apnea index; COPD; Chronic Obstructive Pulmonary Disease.
Statistics presented as Mean ± SD, Median [P25, P75], N (column %).
p-values: a1=t-test, a2=Satterthwaite t-test, b=Wilcoxon Rank Sum test, c=Pearson's chi-square test, d=Fisher's Exact test

© 2021 Pena Orbea C et al. *JAMA Network Open.*

eTable 2 shows a summary of demographics and clinical characteristics by each level of the WHO-7 COVID-19 ordinal scale

**eTable 2.** Data Summary by WHO-7 Ordinal Scale Clinical Outcome Level								
**Factor**	Total (N=1,935)	1.Not Hospitalized (N=1,382)	2.Hospitalized (N=170)	3.Hosp., Supplemental Oxygen (N=191)	4.Hosp. Hi-flow, Non-Inv. Mech. Vent. (N=106)	5.Hosp. ECMO, Inv. Mech. Vent. (N=43)	6.Death (N=43)	p-value
Age (yrs)	56.4 ± 14.5	53.5 ± 13.8	61.6 ± 15.0	63.4 ± 13.7	63.8 ± 12.1	62.4 ± 11.4	72.9 ± 10.9	<0.001[a]
Gender (Male)	955 (49.4)	684 (49.5)	76 (44.7)	87 (45.5)	58 (54.7)	22 (51.2)	28 (65.1)	0.15[c]
Race								<0.001[c]
White	1,236 (63.9)	927 (67.1)	86 (50.6)	114 (59.7)	57 (53.8)	23 (53.5)	29 (67.4)	
Black or African American	537 (27.8)	327 (23.7)	77 (45.3)	64 (33.5)	42 (39.6)	14 (32.6)	13 (30.2)	
Other	162 (8.4)	128 (9.3)	7 (4.1)	13 (6.8)	7 (6.6)	6 (14.0)	1 (2.3)	
Healthcare system site								0.01[d]
Cleveland Clinic Ohio	1,870 (96.6)	1,339 (96.9)	167 (98.2)	184 (96.3)	101 (95.3)	40 (93.0)	39 (90.7)	
Cleveland Clinic Florida	65 (3.4)	43 (3.1)	3 (1.8)	7 (3.7)	5 (4.7)	3 (7.0)	4 (9.3)	
BMI	35.9 ± 9.2	35.8 ± 9.2	35.1 ± 8.3	36.7 ± 9.6	38.0 ± 10.6	35.6 ± 9.0	33.4 ± 8.0	0.04[a]
Coronary Artery Disease	335 (17.3)	167 (12.1)	40 (23.5)	52 (27.2)	39 (36.8)	14 (32.6)	23 (53.5)	<0.001[c]
Hypertension	1,157 (59.8)	728 (52.7)	124 (72.9)	139 (72.8)	87 (82.1)	38 (88.4)	41 (95.3)	<0.001[c]
Heart failure	256 (13.2)	101 (7.3)	39 (22.9)	45 (23.6)	30 (28.3)	16 (37.2)	25 (58.1)	<0.001[c]
Asthma	506 (26.1)	332 (24.0)	49 (28.8)	61 (31.9)	37 (34.9)	12 (27.9)	15 (34.9)	0.02[c]
COPD/emphysema	275 (14.2)	120 (8.7)	41 (24.1)	47 (24.6)	30 (28.3)	19 (44.2)	18 (41.9)	<0.001[c]
Cancer	313 (16.2)	199 (14.4)	40 (23.5)	31 (16.2)	19 (17.9)	6 (14.0)	18 (41.9)	<0.001[c]
Diabetes	613 (31.7)	345 (25.0)	75 (44.1)	90 (47.1)	55 (51.9)	24 (55.8)	24 (55.8)	<0.001[c]
Smoking								<0.001[c]
• No	1,265 (65.4)	931 (67.4)	117 (68.8)	120 (62.8)	54 (50.9)	26 (60.5)	17 (39.5)	
• Current Smoker	100 (5.2)	78 (5.6)	6 (3.5)	7 (3.7)	4 (3.8)	3 (7.0)	2 (4.7)	
• Former Smoker	570 (29.5)	373 (27.0)	47 (27.6)	64 (33.5)	48 (45.3)	14 (32.6)	24 (55.8)	
COVID registry: Smoking pack years								<0.001[b]
• 0.Never	1,520 (78.6)	1,127 (81.5)	133 (78.2)	136 (71.2)	68 (64.2)	31 (72.1)	25 (58.1)	

smoked								
• 0-<10	140 (7.2)	105 (7.6)	11 (6.5)	12 (6.3)	8 (7.5)	2 (4.7)	2 (4.7)	
• 10-<30	152 (7.9)	93 (6.7)	14 (8.2)	23 (12.0)	10 (9.4)	6 (14.0)	6 (14.0)	
• 30+	123 (6.4)	57 (4.1)	12 (7.1)	20 (10.5)	20 (18.9)	4 (9.3)	10 (23.3)	
COVID symptomatic	727 (37.6)	441 (31.9)	77 (45.3)	91 (47.6)	60 (56.6)	24 (55.8)	34 (79.1)	<0.001[c]
Number of COVID symptoms*	0.00 [0.00, 3.0]	0.00 [0.00, 2.0]	0.00 [0.00, 3.0]	0.00 [0.00, 5.0]	1.00 [0.00, 4.0]	2.0 [0.00, 5.0]	2.0 [1.00, 4.0]	<0.001[b]
COVID test timing								<0.001[c]
• March – June 2020	363 (18.8)	210 (15.2)	37 (21.8)	40 (20.9)	29 (27.4)	19 (44.2)	28 (65.1)	
• July – November 2020	1,572 (81.2)	1,172 (84.8)	133 (78.2)	151 (79.1)	77 (72.6)	24 (55.8)	15 (34.9)	

COVID-19 systems: cough, fever, fatigue, sputum production, flu like symptoms, SOB, diarrhea, loss of appetite, vomiting).
Statistics presented as Mean ± SD, Median [P25, P75], N (column %).
p-values: a=ANOVA, b=Kruskal-Wallis test, c=Pearson's chi-square test, d=Fisher's Exact test.

© 2021 Pena Orbea C et al. *JAMA Network Open.*

Sleep Apnea Measures and SARS-CoV-2 Positivity

eTable 3 provides results of propensity score overlap weighting of sleep apnea and sleep-related hypoxia measures and SARS-CoV-2 positivity.

**eTable 3.** Logistic Models for Association Between SARS-CoV-2 Test Outcome and Sleep Apnea Measures Using Propensity Score Overlap Weighting (n=5402)			
Independent variable	Multivariable after adjustment of age, sex, race, BMI, comorbidities*, healthcare system site		
	OR (95%CI)	p-value	E value
Sleep Disordered Breathing Frequency Measure (AHI)			
AHI, 5 event/hr increment	1.00 (0.99 - 1.02)	0.61	1.00
AHI⩾15 vs. <15	1.09 (0.91 - 1.31)	0.34	1.26
AHI categories: 5-14 vs. 0-5	0.98 (0.76 - 1.25)	0.86	1.11
15-30 vs. 0-5	1.03 (0.78 - 1.37)	0.82	1.14
30+ vs. 0-5	1.11 (0.84 - 1.46)	0.46	1.29
Sleep-Related Hypoxia Measures			
TST<90, median >1.5% vs. ⩽1.5%	1.01 (0.84 - 1.20)	0.94	1.08
TST<90, quartiles 0.1-1.5 vs. 0-0.1	1.14 (0.89, 1.44)	0.30	1.34
1.5-11.8 vs. 0-0.1	1.07 (0.84 - 1.36)	0.58	1.22
11.8-100 vs. 0-0.1	1.07 (0.84 - 1.37)	0.57	1.22
Mean SaO2, 5% increment	1.12 (0.97 - 1.31)	0.13	1.31
SaO2 nadir, 5% increment	0.99 (0.93 - 1.04)	0.62	1.08

Abbreviations: AHI: apnea hypopnea index; BMI: Body mass index; OR: Odds ratio; CI: Confidence Interval; TST<90 (total sleep time spent with SaO2<90%)
*Comorbidities: diabetes, hypertension, coronary artery disease, heart failure, cancer, asthma, COPD/ emphysema and smoking pack years

Association of Sleep Apnea Measures and COVID-19 Clinical outcomes

e-Table 4 demonstrates the association sleep-related hypoxia measures with increasing WHO-7 COVID-19 ordinal scale outcome in patients without PAP use on COVID-19 testing. In adjusted analyses, median TST<90 remained significantly associated with increasing WHO-7 COVID-19 ordinal scale and increased hospitalization and death from the time of COVID-19 symptom onset. **(e-Table 5.)** Similar findings were observed in the other subgroups of patients using the 3% hypopnea rule and undergoing in-laboratory sleep study only (**e-Tables 6,7,8 and 9**)

**eTable 4.** Ordinal Logistic Models of WHO-7 Outcome Before and After Adjustment, for Adult Patients Without PAP Use on COVID-19 Testing (N=1610)						
Independent variable	Model 1: Univariable		Model 2: Multivariable after adjustment of age, sex, race and BMI		Model 3: Multivariable after adjustment of age, sex, race, BMI, comorbidities, Healthcare system site	
	OR (95%CI)	p-value	OR (95%CI)	p-value	OR (95%CI)	p-value
Sleep Disordered Breathing Frequency Measure (AHI)						
AHI, 5 event/hr increment	1.03 (1.01 - 1.05)	*0.002*	1.00 (0.98 - 1.02)	0.84	0.99 (0.97 - 1.02)	0.63
AHI⩾15 vs. <15	1.41 (1.14 - 1.75)	*0.002*	1.03 (0.81 - 1.31)	0.83	1.01 (0.79 - 1.30)	0.93
AHI categories:<br>5-14 vs. 0-5	1.77 (1.27 - 2.48)	*<0.001*	1.33 (0.93 - 1.92)	0.12	1.25 (0.86, -1.82)	0.24
15-30 vs. 0-5	1.95 (1.36 - 2.80)	*<0.001*	1.37 (0.92 - 2.03)	0.12	1.39(0.93 - 2.09)	0.11
30+ vs. 0-5	2.05 (1.48 - 2.85)	*<0.001*	1.18 (0.81 - 1.73)	0.39	1.06 (0.72 - 1.56)	0.78
Sleep-Related Hypoxia Measures						
TST<90, 5% increment	1.06 (1.03 - 1.09)	*<0.001*	1.04 (1.01 - 1.07)	*0.005*	1.02 (0.99 - 1.05)	0.15
TST<90, median:<br>>1.8% vs. ⩽1.8%	1.94 (1.55 - 2.42)	*<0.001*	1.50 (1.17 - 1.92)	*0.001*	1.33 (1.03 - 1.72)	*0.03*
TST<90, quartiles:<br>0.1-1.8 vs. 0-0.1	1.47 (1.05 - 2.06)	*0.02*	1.12 (0.78 - 1.60)	0.55	1.05 (0.72 - 1.51)	0.81
1.8-12.8 vs. 0-0.1	2.17 (1.58 - 2.98)	*<0.001*	1.42 (1.00 - 2.02)	0.05	1.33 (0.93 - 1.91)	0.12
12.8-100 vs. 0-0.1	2.54 (1.85 - 3.48)	*<0.001*	1.81 (1.27 - 2.58)	*0.001*	1.40 (0.97 - 2.03)	0.07
Mean SaO2, 5% increment	0.58 (0.48 - 0.71)	*<0.001*	0.66 (0.52 - 0.82)	*<0.001*	0.75 (0.60 - 0.95)	*0.02*
SaO2 nadir, 5% increment	0.85 (0.80 - 0.91)	*<0.001*	0.91 (0.85 - 0.98)	*0.01*	0.93 (0.86 - 1.00)	0.06
TST<90, 5% increment	1.06 (1.03 - 1.09)	*<0.001*	1.04 (1.01 - 1.07)	*0.005*	1.02 (0.99 - 1.05)	0.15

Abbreviations: PAP: Positive airway pressure, BMI: Body mass index, AHI: apnea hypopnea index; TST<90 (total sleep time spent with SaO2<90%), HR: Hazards Ratio, CI: Confidence interval.

*Comorbidities: diabetes, hypertension, coronary artery disease, heart failure, cancer, asthma, COPD/ emphysema and smoking pack years

© 2021 Pena Orbea C et al. *JAMA Network Open.*

**eTable 5.** Cox Proportional Hazard Models of Hospitalization and Death Without PAP Use on COVID-19 Testing (N=1601)						
Independent variable	Model 1: Univariable		Model 2: Multivariable after adjustment of age, sex, race and BMI		Model 3: Multivariable after adjustment of age, sex, race, BMI, comorbidities, health care system site	
	HR (95%CI)	p-value	HR (95%CI)	p-value	HR (95%CI)	p-value
Sleep Disordered Breathing Frequency Measure (AHI)						
AHI, 5 event/hr increment	1.02 (1.01 - 1.04)	*0.003*	1.01 (0.99 - 1.02)	0.47	1.00 (0.98 - 1.02)	0.99
AHI⩾15 vs. <15	1.27 (1.06 - 1.53)	*0.01*	1.00 (0.82 - 1.22)	0.97	0.96 (0.78 - 1.17)	0.67
AHI categories:<br>5-14 vs. 0-5	1.61 (1.20 - 2.17)	*0.002*	1.24 (0.92 - 1.67)	0.17	1.22 (0.90 - 1.66)	0.19
15-30 vs. 0-5	1.63 (1.18 - 2.24)	*0.003*	1.15 (0.83 - 1.60)	0.41	1.13 (0.81 - 1.58)	0.48
30+ vs. 0-5	1.78 (1.33 - 2.38)	*<0.001*	1.17 (0.86 - 1.60)	0.32	1.09 (0.79 - 1.50)	0.60
Sleep-Related Hypoxia Measures						
TST<90, median:<br>>1.8% vs. ⩽1.8%	1.66 (1.37 - 2.01)	*<0.001*	1.37 (1.11 - 1.67)	*0.003*	1.27 (1.03 - 1.56)	*0.024*
TST<90, quartiles:<br>0.1-1.8 vs. 0-0.1	1.39 (1.03 - 1.87)	*0.03*	1.00 (0.74 - 1.35)	0.99	0.99 (0.72 - 1.34)	0.92
1.8-12.8 vs. 0-0.1	1.87 (1.41 - 2.47)	*<0.001*	1.27 (0.95 - 1.70)	0.11	1.27 (0.94 - 1.71)	0.11
12.8-100 vs. 0-0.1	2.03 (1.54 - 2.69)	*<0.001*	1.47 (1.10 - 1.98)	*0.010*	1.24 (0.92 - 1.68)	0.16
Mean SaO2, 5% increment	0.67 (0.57 - 0.78)	*<0.001*	0.73 (0.61 - 0.86)	*<0.001*	0.81 (0.68 - 0.97)	*0.02*
SaO2 nadir, 5% increment	0.89 (0.84 - 0.94)	*<0.001*	0.93 (0.88 - 0.99)	*0.024*	0.95 (0.89 - 1.01)	0.09
TST<90, 5% increment	0.89 (0.85 - 0.94)	*<0.001*	0.94 (0.88 - 1.00)	*0.037*	0.95 (0.90 - 1.02)	0.14

Abbreviations: PAP: Positive airway pressure, BMI: Body mass index, AHI: apnea hypopnea index; TST<90 (total sleep time spent with SaO2<90%),HR: Hazards Ratio, CI: Confidence interval

*Comorbidities: diabetes, hypertension, coronary artery disease, heart failure, cancer, asthma, COPD/ emphysema and smoking pack years

© 2021 Pena Orbea C et al. *JAMA Network Open.*

**eTable 6.** Ordinal Logistic Models of WHO-7 Outcome Using the 3% Hypopnea Rule (n=1336)						
Independent variable	Model 1: Univariable		Model 2: Multivariable after adjustment of age, sex, race and BMI		Model 3: Multivariable after adjustment of age, sex, race, BMI, comorbidities, clinic site	
	OR (95%CI)	p-value	OR (95%CI)	p-value	OR (95%CI)	p-value
Sleep Disordered Breathing Frequency Measure (AHI)						
AHI, 5 event/hr increment	1.03(1.01 - 1.05)	*0.007*	1.01(0.98 - 1.03)	0.60	1.01(0.98 - 1.03)	0.57
AHI⩾15 vs. <15	1.48(1.14 - 1.91)	*0.003*	1.02(0.77 - 1.36)	0.88	1.05(0.78 - 1.41)	0.77
AHI categories:<br>5-14 vs. 0-5	1.67(1.08 - 2.60)	*0.02*	1.32(0.83 - 2.11)	0.25	1.20(0.74 - 1.95)	0.45
15-30 vs. 0-5	1.94(1.23 - 3.06)	*0.004*	1.27(0.78 - 2.07)	0.33	1.22(0.74 - 2.02)	0.43
30+ vs. 0-5	2.19(1.43 - 3.34)	*<0.001*	1.24(0.77 - 2.00)	0.38	1.18(0.72 - 1.93)	0.51
Sleep-Related Hypoxia Measures						
TST<90, median:<br>>1.8% vs. ⩽1.8%	2.24(1.72 - 2.92)	*<0.001*	1.81(1.35 - 2.43)	*<0.001*	1.78(1.32 - 2.41)	*<0.001*
TST<90 quartiles:<br>0.1-1.8 vs. 0-0.1	1.67(1.10 - 2.53)	*0.02*	1.32(0.85 - 2.04)	0.22	1.30(0.83 - 2.04)	0.25
1.8-12.8 vs. 0-0.1	2.71(1.83 - 4.01)	*<0.001*	1.91(1.25 - 2.92)	*0.003*	1.99(1.28 - 3.09)	*0.002*
12.8-100 vs. 0-0.1	3.13(2.13 - 4.61)	*<0.001*	2.39(1.55 - 3.67)	*<0.001*	2.18(1.39 - 3.40)	*<0.001*
Mean SaO2, 5% increment	0.55(0.44 - 0.69)	*<0.001*	0.57(0.44 - 0.75)	*<0.001*	0.62(0.47 - 0.81)	*<0.001*
SaO2 nadir, 5% increment	0.85(0.79 - 0.92)	*<0.001*	0.90(0.83 - 0.98)	*0.02*	0.91(0.83 - 1.00)	*0.04*
TST<90, 5% increment	1.07(1.04 - 1.10)	*<0.001*	1.05(1.02 - 1.09)	*<0.001*	1.04(1.01 - 1.08)	*0.01*

Abbreviations: BMI: Body mass index, AHI: apnea hypopnea index; TST<90 (total sleep time spent with SaO2<90%), HR: Hazards Ratio, CI: Confidence interval

*Comorbidities: diabetes, hypertension, coronary artery disease, heart failure, cancer, asthma, COPD/ emphysema and smoking pack years

© 2021 Pena Orbea C et al. *JAMA Network Open.*

eTable 7. Cox Proportional Hazard Models of Hospitalization and Death Using 3% Hypopnea Rule (N=1336)

Independent variable	Model 1: Univariable		Model 2: Multivariable after adjustment of age, sex, race and BMI		Model 3: Multivariable after adjustment of age, sex, race, BMI, comorbidities, health care system site	
	HR (95%CI)	p-value	HR (95%CI)	p-value	HR (95%CI)	p-value
Sleep Disordered Breathing Frequency Measure (AHI)						
AHI, 5 event/hr increment	1.02 (1.01 - 1.04)	*0.006*	1.01 (0.99 - 1.03)	0.25	1.01 (0.99 - 1.03)	0.23
AHI⩾15 vs. <15	1.37 (1.09 - 1.72)	*0.006*	1.03 (0.81 - 1.31)	0.81	1.00 (0.78 - 1.27)	0.97
AHI categories: 5-14 vs. 0-5	1.55 (1.04 - 2.32)	*0.03*	1.30 (0.87 - 1.95)	0.20	1.27 (0.85 - 1.92)	0.25
15-30 vs. 0-5	1.70 (1.12 - 2.56)	*0.012*	1.16 (0.76 - 1.77)	0.49	1.08 (0.70 - 1.66)	0.72
30+ vs. 0-5	1.95 (1.33 - 2.86)	*<0.001*	1.31 (0.87 - 1.96)	0.20	1.28 (0.84 - 1.94)	0.25
Sleep-Related Hypoxia Measures						
TST<90 by median: >1.8% vs. ⩽1.8%	1.92 (1.51 - 2.43)	*<0.001*	1.61 (1.26 - 2.06)	*<0.001*	1.59 (1.23 - 2.04)	*<0.001*
TST<90 quartiles: 0.1-1.8 vs. 0-0.1	1.57 (1.08 - 2.30)	*0.02*	1.20 (0.82 - 1.76)	0.35	1.22 (0.83 - 1.81)	0.32
1.8-12.8 vs. 0-0.1	2.35 (1.65 - 3.34)	*<0.001*	1.69 (1.17 - 2.43)	*0.005*	1.79 (1.23 - 2.60)	*0.002*
12.8-100 vs. 0-0.1	2.50 (1.76 - 3.54)	*<0.001*	1.91 (1.32 - 2.77)	*<0.001*	1.78 (1.22 - 2.60)	*0.003*
Mean SaO2, 5% increment	0.65 (0.54 - 0.79)	*<0.001*	0.66 (0.53 - 0.81)	*<0.001*	0.70 (0.57 - 0.87)	*0.001*
SaO2 nadir, 5% increment	0.88 (0.83 - 0.94)	*<0.001*	0.92 (0.86 - 0.99)	*0.024*	0.93 (0.86 - 1.00)	*0.05*
TST<90, 5% increment	1.05 (1.03 - 1.08)	*<0.001*	1.04 (1.02 - 1.07)	*<0.001*	1.03 (1.01 - 1.06)	*0.013*

Abbreviations: AHI: apnea hypopnea index; BMI: Body Mass Index, TST<90 (total sleep time spent with SaO2<90%) HR: Hazards Ratio, CI: Confidence interval

*Comorbidities: diabetes, hypertension, coronary artery disease, heart failure, cancer, asthma, COPD/ emphysema and smoking pack years

© 2021 Pena Orbea C et al. *JAMA Network Open.*

eTable 8. Ordinal Logistic Models of WHO-7 Outcome on Patients Having Polysomnogram (n=1538)

Independent variable	Model 1: Univariable		Model 2: Multivariable after adjustment of age, sex, race and BMI		Model 3: Multivariable after adjustment of age, sex, race, BMI, comorbidities, clinic site	
	OR (95%CI)	p-value	OR (95%CI)	p-value	OR (95%CI)	p-value
Sleep Disordered Breathing Frequency Measure (AHI)						
AHI, 5 event/hr increment	1.02 (1.01 - 1.04)	0.006	1.00 (0.98 - 1.03)	0.64	1.00 (0.98 - 1.02)	0.90
AHI⩾15 vs. <15	1.42 (1.15 - 1.76)	0.001	1.03 (0.81 - 1.31)	0.82	0.99 (0.77 - 1.27)	0.93
AHI categories: 5-14 vs. 0-5	1.95 (1.36 - 2.79)	<0.001	1.47 (0.99 - 2.16)	0.05	1.39 (0.93 - 2.08)	0.10
15-30 vs. 0-5	2.09 (1.42 - 3.07)	<0.001	1.41 (0.93 - 2.15)	0.11	1.36 (0.88 - 2.08)	0.17
30+ vs. 0-5	2.28 (1.61 - 3.24)	<0.001	1.34 (0.89 - 2.00)	0.16	1.21 (0.80 - 1.83)	0.37
Sleep-Related Hypoxia Measures						
TST<90, median: >1.8% vs. ≤1.8%	2.21 (1.78 - 2.75)	<0.001	1.76 (1.39- 2.25)	<0.001	1.57 (1.22 - 2.01)	<0.001
TST<90 quartiles: 0.1-1.8 vs. 0-0.1	1.65 (1.19 - 2.28)	0.003	1.25 (0.88 - 1.76)	0.22	1.21 (0.85 - 1.73)	0.29
1.8-12.8 vs. 0-0.1	2.54 (1.85 - 3.48)	<0.001	1.73 (1.23- 2.45)	0.002	1.65 (1.16 - 2.36)	0.005
12.8-100 vs. 0-0.1	3.34 (2.42 - 4.62)	<0.001	2.45 (1.71 - 3.53)	<0.001	1.91 (1.31 - 2.79)	<0.001
Mean SaO2, 5% increment	0.56 (0.46 - 0.67)	<0.001	0.62 (0.50 - 0.77)	<0.001	0.72 (0.57 - 0.90)	0.004
SaO2 nadir, 5% increment	0.84 (0.79 - 0.90)	<0.001	0.90 (0.84 - 0.97)	0.004	0.92 (0.85 - 0.99)	0.02
TST<90, 5% increment	1.09 (1.06 - 1.12)	<0.001	1.07 (1.04 - 1.10)	<0.001	1.05 (1.02 - 1.08)	0.003

Abbreviations: BMI: Body mass index, AHI: apnea hypopnea index; TST<90 (total sleep time spent with SaO2<90%), HR: Hazards Ratio, CI: Confidence interval.

*Comorbidities: diabetes, hypertension, coronary artery disease, heart failure, cancer, asthma, COPD/ emphysema and smoking pack years

© 2021 Pena Orbea C et al. *JAMA Network Open.*

**eTable 9.** Cox Proportional Hazard Models of Hospitalization and Death on Patients Having Polysomnogram (N=1586)						
**Independent variable**	Model 1: Univariable		Model 2: Multivariable after adjustment of age, sex, race and BMI		**Model 3: Multivariable after adjustment of age, sex, race, BMI, comorbidities, health care system site**	
	HR (95%CI)	p-value	HR (95%CI)	p-value	**HR (95%CI)**	**p-value**
Sleep Disordered Breathing Frequency Measure (AHI)						
AHI, 5 event/hr increment	1.02 (1.01 - 1.03)	0.006	1.01 (0.99 - 1.02)	0.46	1.00 (0.98 - 1.02)	0.89
AHI⩾15 vs. <15	1.30 (1.08 - 1.57)	0.005	1.00 (0.82 - 1.22)	0.99	0.96 (0.79 - 1.18)	0.73
AHI categories: 5-14 vs. 0-5	1.68 (1.22 - 2.31)	0.001	1.26 (0.91 - 1.75)	0.16	1.23 (0.88 - 1.71)	0.22
15-30 vs. 0-5	1.72 (1.23 - 2.42)	0.002	1.15 (0.81 - 1.63)	0.45	1.11 (0.78 - 1.59)	0.56
30+ vs. 0-5	1.92 (1.41 - 2.61)	<0.001	1.22 (0.87 - 1.72)	0.25	1.14 (0.80 - 1.61)	0.47
Sleep-Related Hypoxia Measures						
TST<90 by median: >1.8% vs. ⩽1.8%	1.81 (1.50 - 2.19)	<0.001	1.47 (1.20, 1.80)	<0.001	1.35 (1.10 - 1.65)	0.004
TST<90 quartiles: 0.1-1.8 vs. 0-0.1	1.54 (1.15 - 2.06)	0.003	1.13 (0.84, 1.52)	0.42	1.12 (0.83 - 1.51)	0.45
1.8-12.8 vs. 0-0.1	2.10 (1.59 - 2.77)	<0.001	1.45 (1.08, 1.94)	0.01	1.43 (1.07, -1.91)	0.02
12.8-100 vs. 0-0.1	2.51 (1.89 - 3.32)	<0.001	1.78 (1.31, 2.41)	<0.001	1.47 (1.08 - 1.99)	0.01
Mean SaO2, 5% increment	0.67 (0.58 - 0.77)	<0.001	0.73 (0.62, 0.86)	<0.001	0.82 (0.70 - 0.97)	0.02
SaO2 nadir, 5% increment	0.88 (0.84 - 0.93)	<0.001	0.93 (0.88 - 0.99)	0.02	0.94 (0.89 - 1.00)	0.05
TST<90, 5% increment	1.06 (1.04- 1.09)	<0.001	1.05 (1.03 - 1.07)	<0.001	1.03 (1.01 - 1.06)	0.006

Abbreviations: AHI: apnea hypopnea index; BMI: Body Mass Index, TST<90 (total sleep time spent with SaO2<90%), HR: Hazards Ratio, CI: Confidence interval

*Comorbidities: diabetes, hypertension, coronary artery disease, heart failure, cancer, asthma, COPD/ emphysema and smoking pack years

© 2021 Pena Orbea C et al. *JAMA Network Open.*

**eTable 10.** Ordinal Logistic Models of WHO-7 Outcome on Patients Who Had a Sleep Study Within 5 Years of the COVID-19 Test (n=1017)						
Independent variable	Model 1: Univariable		Model 2: Multivariable after adjustment of age, sex, race and BMI		Model 3: Multivariable after adjustment of age, sex, race, BMI, comorbidities, clinic site	
	OR (95%CI)	p-value	OR (95%CI)	p-value	OR (95%CI)	**p-value**
Sleep Disordered Breathing Frequency Measure (AHI)						
AHI, 5 event/hr increment	1.01 (0.99 - 1.03)	0.34	0.98 (0.96 - 1.01)	0.27	0.98 (0.95 - 1.01)	0.15
AHI≥15 vs. <15	1.31 (1.00 - 1.71)	0.05	0.96 (0.71 - 1.31)	0.81	0.94 (0.68 - 1.28)	0.68
AHI categories: 5-14 vs. 0-5	1.61 (1.03 - 2.51)	0.04	1.11 (0.69 -1.80)	0.67	1.16 (0.71 - 1.91)	0.55
15-30 vs. 0-5	1.86 (1.16 - 2.97)	0.01	1.18 (0.70 - 1.97)	0.54	1.21 (0.71 - 2.05)	0.48
30+ vs. 0-5	1.78 (1.15 - 2.76)	0.01	0.96 (0.58 - 1.58)	0.86	0.94 (0.56 - 1.59)	0.83
Sleep-Related Hypoxia Measures						
TST<90, median: >1.8% vs. ≤1.8%	1.74 (1.32 - 2.28)	<0.001	1.28 (0.94 - 1.74)	0.11	1.16 (0.85 - 1.59)	0.36
TST<90 quartiles: 0.1-1.8 vs. 0-0.1	2.23 (1.46 -3.40)	<0.001	1.64 (1.04 - 2.57)	0.033	1.73 (1.09 - 2.75)	0.02
1.8-12.8 vs. 0-0.1	2.64 (1.73 - 4.01)	<0.001	1.56 (0.99 - 2.48)	0.057	1.62 (1.01 - 2.59)	0.05
12.8-100 vs. 0-0.1	2.23 (1.46 - 3.40)	<0.001	1.64 (1.04 - 2.57)	0.033	1.73 (1.09 - 2.75)	0.02
Mean SaO2, 5% increment	0.65 (0.51 - 0.81)	<0.001	0.72 (0.54 - 0.94)	0.017	0.81 (0.61 - 1.07)	0.14
SaO2 nadir, 5% increment	0.86 (0.79 - 0.93)	<0.001	0.92 (0.84 - 1.01)	0.01	0.94 (0.85 - 1.04)	0.22
TST<90, 5% increment	1.04 (1.01 - 1.07)	0.01	1.03 (1.00 - 1.07)	0.08	1.02 (0.98 - 1.05)	0.41

Abbreviations: BMI: Body mass index, AHI: apnea hypopnea index; TST<90 (total sleep time spent with SaO2<90%), HR: Hazards Ratio, CI: Confidence interval.
*Comorbidities: diabetes, hypertension, coronary artery disease, heart failure, cancer, asthma, COPD/ emphysema and smoking pack years

© 2021 Pena Orbea C et al. *JAMA Network Open.*

**eTable 11.** Cox Proportional Hazard Models of Hospitalization and Death on Patients Who Had a Sleep Study Within 5 Years of the COVID-19 Test (n=1013)						
Independent variable	Model 1: Univariable		Model 2: Multivariable after adjustment of age, sex, race and BMI		Model 3: Multivariable after adjustment of age, sex, race, BMI, comorbidities, health care system site	
	HR (95%CI)	p-value	HR (95%CI)	p-value	HR (95%CI)	p-value
Sleep Disordered Breathing Frequency Measure (AHI)						
AHI, 5 event/hr increment	1.01 (0.99 - 1.03)	0.38	0.99 (0.97 - 1.01)	0.36	0.99 (0.97 - 1.01)	0.29
AHI⩾15 vs. <15	1.19 (0.94 - 1.50)	0.14	0.96 (0.75 - 1.23)	0.73	0.98 (0.76 - 1.26)	0.88
AHI categories: 5-14 vs. 0-5	1.50 (1.02 - 2.23)	0.04	1.06 (0.71 - 1.58)	0.78	1.05 (0.70 - 1.57)	0.83
15-30 vs. 0-5	1.56 (1.03 - 2.37)	0.04	1.04 (0.68 - 1.60)	0.86	1.07 (0.69 - 1.65)	0.76
30+ vs. 0-5	1.59 (1.08 - 2.34)	0.02	0.97 (0.64 - 1.48)	0.89	0.97 (0.64 - 1.50)	0.91
Sleep-Related Hypoxia Measures						
TST<90 by median: >1.8% vs. ⩽1.8%	1.53 (1.21 - 1.94)	<0.001	1.21 (0.94 - 1.55)	0.14	1.17 (0.90 - 1.51)	0.25
TST<90 quartiles: 0.1-1.8 vs. 0-0.1	2.08 (1.43 - 3.03)	<0.001	1.52 (1.03 - 2.23)	0.033	1.68 (1.14 - 2.47)	0.009
1.8-12.8 vs. 0-0.1	2.29 (1.58 - 3.33)	<0.001	1.45 (0.99 - 2.15)	0.059	1.60 (1.08 - 2.38)	0.019
12.8-100 vs. 0-0.1	2.27 (1.57 - 3.27)	<0.001	1.69 (1.15 - 2.50)	0.008	1.59 (1.07 - 2.38)	0.023
Mean SaO2, 5% increment	0.75 (0.62 - 0.90)	0.002	0.83 (0.67 - 1.02)	0.08	0.90 (0.73 - 1.12)	0.35
SaO2 nadir, 5% increment	0.89 (0.84 - 0.95)	<0.001	0.94 (0.87 - 1.02)	0.12	0.95 (0.88 - 1.02)	0.19
TST<90, 5% increment	1.03 (1.00 - 1.05)	0.03	1.02 (1.00 - 1.05)	0.09	1.01 (0.99 - 1.04)	0.35

Abbreviations: AHI: apnea hypopnea index; BMI: Body Mass Index, TST<90 (total sleep time spent with SaO2<90%), HR: Hazards Ratio, CI: Confidence interval
*Comorbidities: diabetes, hypertension, coronary artery disease, heart failure, cancer, asthma, COPD/ emphysema and smoking pack years

© 2021 Pena Orbea C et al. *JAMA Network Open.*

eTable 12. Ordinal Logistic Models of WHO-7 Outcome on Patients Who Had a Sleep Study More Than 5 Years of the COVID-19 Test (n=918)

Independent variable	Model 1: Univariable		Model 2: Multivariable after adjustment of age, sex, race and BMI		Model 3: Multivariable after adjustment of age, sex, race, BMI, comorbidities, clinic site	
	OR (95%CI)	p-value	OR (95%CI)	p-value	OR (95%CI)	p-value
Sleep Disordered Breathing Frequency Measure (AHI)						
AHI, 5 event/hr increment	1.03 (1.01 - 1.06)	0.003	1.02 (0.99, 1.04)	0.24	1.01 (0.98, 1.03)	0.65
AHI⩾15 vs. <15	1.52 (1.14 - 2.02)	0.01	1.07 (0.77, 1.47)	0.70	1.02 (0.73, 1.42)	0.93
AHI categories: 5-14 vs. 0-5	1.71 (1.08 - 2.72)	0.02	1.36 (0.82, 2.24)	0.23	1.15 (0.69 - 1.93)	0.60
15-30 vs. 0-5	1.91 (1.16 - 3.13)	0.011	1.31 (0.77, 2.24)	0.32	1.22 (0.70 - 2.12)	0.48
30+ vs. 0-5	2.26 (1.45 - 3.53)	<0.001	1.32 (0.79, 2.22)	0.29	1.05 (0.61 - 1.79)	0.86
Sleep-Related Hypoxia Measures						
TST<90, median: >1.8% vs. ⩽1.8%	2.23 (1.65 - 3.01)	<0.001	1.83 (1.32 - 2.55)	<0.001	1.61 (1.14 - 2.26)	0.006
TST<90 quartiles: 0.1-1.8 vs. 0-0.1	1.09 (0.68 - 1.73)	0.72	0.92 (0.56 - 1.51)	0.75	0.84 (0.50 - 1.40)	0.50
1.8-12.8 vs. 0-0.1	2.14 (1.40 - 3.27)	<0.001	1.63 (1.03 - 2.60)	0.04	1.45 (0.90 - 2.34)	0.12
12.8-100 vs. 0-0.1	2.55 (1.67 - 3.91)	<0.001	1.92 (1.20 - 3.09)	0.007	1.48 (0.90 - 2.42)	0.12
Mean SaO2, 5% increment	0.54 (0.41 - 0.71)	<0.001	0.62 (0.46 - 0.84)	0.002	0.70 (0.51 - 0.97)	0.03
SaO2 nadir, 5% increment	0.87 (0.79 - 0.95)	0.001	0.91 (0.83 - 1.01)	0.07	0.94 (0.85- 1.04)	0.21
TST<90, 5% increment	1.07 (1.04 - 1.11)	<0.001	1.05 (1.01 - 1.08)	0.02	1.03 (0.99 - 1.07)	0.21

Abbreviations: BMI: Body mass index, AHI: apnea hypopnea index; TST<90 (total sleep time spent with SaO2<90%), HR: Hazards Ratio, CI: Confidence interval.

*Comorbidities: diabetes, hypertension, coronary artery disease, heart failure, cancer, asthma, COPD/ emphysema and smoking pack years

© 2021 Pena Orbea C et al. *JAMA Network Open.*

**eTable 13.** Cox Proportional Hazard Models of Hospitalization and Death on Patients Who Had a Sleep Study More Than 5 Years of the COVID-19 Test (n=912)						
Independent variable	Model 1: Univariable		Model 2: Multivariable after adjustment of age, sex, race and BMI		Model 3: Multivariable after adjustment of age, sex, race, BMI, comorbidities, health care system site	
	HR (95%CI)	p-value	HR (95%CI)	p-value	HR (95%CI)	p-value
Sleep Disordered Breathing Frequency Measure (AHI)						
AHI, 5 event/hr increment	1.03 (1.01 - 1.05)	0.002	1.02 (1.00 - 1.04)	0.06	1.02 (0.99- 1.04)	0.21
AHI⩾15 vs. <15	1.44 (1.12 - 1.85)	0.005	1.09 (0.83- 1.43)	0.55	1.01 (0.77 - 1.33)	0.93
AHI categories:						
5-14 vs. 0-5	1.49 (0.98 - 2.26)	0.06	1.19 (0.78 - 1.81)	0.42	1.17 (0.76 - 1.80)	0.48
15-30 vs. 0-5	1.67 (1.07 - 2.59)	0.02	1.11 (0.71 - 1.75)	0.65	1.09 (0.69 - 1.73)	0.72
30+ vs. 0-5	1.95 (1.32 - 2.90)	<0.001	1.31 (0.85 - 2.01)	0.22	1.16 (0.74 - 1.80)	0.52
Sleep-Related Hypoxia Measures						
TST<90 by median: >1.8% vs. ⩽1.8%	1.85 (1.42 - 2.41)	<0.001	1.57 (1.19 - 2.07)	0.001	1.42 (1.07 - 1.87)	0.015
TST<90 quartiles: 0.1-1.8 vs. 0-0.1	1.03 (0.68 - 1.55)	0.91	0.79 (0.52 - 1.21)	0.29	0.75 (0.49 - 1.14)	0.18
1.8-12.8 vs. 0-0.1	1.79 (1.24 - 2.59)	0.002	1.34 (0.91 - 1.96)	0.14	1.25 (0.85 - 1.84)	0.26
12.8-100 vs. 0-0.1	1.97 (1.36 - 2.86)	<0.001	1.45 (0.98 - 2.15)	0.07	1.16 (0.78 - 1.73)	0.46
Mean SaO2, 5% increment	0.62 (0.50 - 0.77)	<0.001	0.65 (0.51 - 0.83)	<0.001	0.71 (0.56 - 0.91)	0.008
SaO2 nadir, 5% increment	0.90 (0.83 - 0.96)	0.003	0.93 (0.86 - 1.01)	0.09	0.94 (0.87 - 1.03)	0.17
TST<90, 5% increment	1.05 (1.02 - 1.08)	<0.001	1.04 (1.01 - 1.07)	0.01	1.02 (0.99 - 1.06)	0.14

Abbreviations: AHI: apnea hypopnea index; BMI: Body Mass Index, TST<90 (total sleep time spent with SaO2<90%),
HR: Hazards Ratio, CI: Confidence interval
*Comorbidities: diabetes, hypertension, coronary artery disease, heart failure, cancer, asthma, COPD/ emphysema and smoking pack years

© 2021 Pena Orbea C et al. *JAMA Network Open.*

Mediation analysis between CRP and sleep related hypoxia measures.

Mediation analysis was performed on the subgroup of patients who had blood sample of C-reactive protein (CRP) on admission. The dependent variable was the dichotomized outcome of high-level WHO-7 COVID-19 scale (any Supplemental Oxygen, High flow/Noninvasive mechanical ventilation, ECMO/Invasive mechanical ventilation, or Death). CRP was assumed to have mediation effect in the causal pathway between high-level WHO-7 COVID-19 scale and sleep indices. To satisfy normal distribution assumption, logarithm transformation was used on CRP. Both outcome model and mediator model were adjusted for demographics, comorbidities, smoking pack year and clinic site. Natural indirect effect was estimated by using PROC CAUSALMED statement in SAS software (version 9.4, Cary, NC).

To assess the mediation effect of CRP (M), the first step was testing relationships between sleep indices (T) and the outcome (Y). If T -> Y is significant, we require significant relationship between CRP and sleep indices (T -> M). Then, the mediation effect, or natural indirect effect, was estimated by a logistic regression model of Y, with both T and M in the model, and comparing with the first model (T -> Y). Based on **e-Table 13** (first models), TST<90 by median, mean oxygen saturation and minimum oxygen saturation met the criteria of significance. Specifically, TST<90 by median was not associated with CRP (p=0.13), while mean oxygen saturation and minimum oxygen saturation had significant associations with CRP (p=0.040, 0.029, respectively). Moreover, both mean oxygen saturation and minimum oxygen saturation had significant natural indirect effect of CRP (mean SaO2: OR 0.89, 95%CI 0.73-0.99, p=0.036; SaO2 nadir: OR 0.95, 95%CI 0.90-1.00, p=0.034). Therefore, CRP played a role of mediator in the causal pathway between high-level WHO-7 COVID-19 scale with mean oxygen saturation and minimum oxygen saturation.

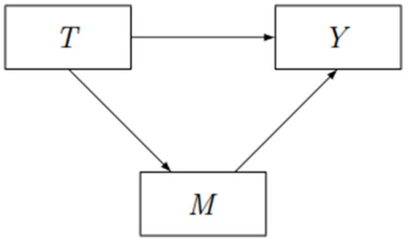

© 2021 Pena Orbea C et al. *JAMA Network Open.*

**eTable 14.** Logistic Models of Any High-Level WHO Scale (N=466)		
Independent variable	Multivariable after adjustment of age, sex, race, BMI, comorbidities*, healthcare system site	
	OR (95%CI)	p-value
Sleep Disordered Breathing Frequency Measure (AHI)		
AHI, 5 event/hr increment	1.02 (0.98 - 1.07)	0.30
AHI⩾15 vs. <15	1.55 (0.98 - 2.46)	0.06
AHI categories:<br>5-14 vs. 0-5	1.03 (0.51 - 2.07)	0.94
15-30 vs. 0-5	1.55 (0.73 - 3.27)	0.26
30+ vs. 0-5	1.61 (0.77 - 3.39)	0.21
Sleep-Related Hypoxia Measures		
TST<90, median:<br>>1.8% vs. ⩽1.8%	2.04 (1.28 - 3.23)	*0.003*
TST<90, quartiles:<br>0.1-1.8 vs. 0-0.1	0.91 (0.47 - 1.76)	0.78
1.8-12.8 vs. 0-0.1	1.53 (0.79 - 2.93)	0.21
12.8-100 vs. 0-0.1	2.74 (1.32 - 5.69)	0.007
Mean SaO2, 5% increment	0.43 (0.26 - 0.70)	*<0.001*
SaO2 nadir, 5% increment	0.84 (0.72 - 0.99)	*0.03*
TST<90, 5% increment	1.06 (1.00 - 1.13)	0.06

Abbreviations: AHI: apnea hypopnea index; TST<90 (total sleep time spent with SaO2<90%)
*Comorbidities: diabetes, hypertension, coronary artery disease, heart failure, cancer, asthma,COPD/ emphysema and smoking pack years.

© 2021 Pena Orbea C et al. *JAMA Network Open.*

OSA and CSA subtype analyses

**eTable 15.** Ordinal Logistic Models of WHO-7 Outcome (n=1935)						
Independent variable	Model 1<br>Univariable		Model 2<br>Multivariable after adjustment of age, sex, race and BMI		Model 3<br>Multivariable after adjustment of age, sex, race, BMI, comorbidities*, healthcare system site	
	OR (95%CI)	p-value	OR (95%CI)	p-value	OR (95%CI)	p-value
Sleep Disordered Breathing Frequency Measure (AHI)						
CAI, 5 event/hr increment	1.10 (1.01 - 1.21)	*0.03*	1.04 (0.95 - 1.15)	0.38	1.01 (0.92 - 1.12)	0.78
OAI, 5 event/hr increment	1.00 (0.94 - 1.08)	0.92	0.93 (0.85 - 1.02)	0.11	0.95 (0.87 - 1.04)	0.23
Sleep-Related Hypoxia Measures						
Max ETCO2, 1 mmHg increment	1.00 (0.98 - 1.03)	0.85	0.99 (0.97 - 1.02)	0.71	0.99 (0.96 - 1.02)	0.57

Abbreviations: AHI: apnea hypopnea index; CAI: Central apnea index; OAI: Obstructive apnea index; BMI; Body mass index; OR: Odds ratio; CI: Confidence Interval; TST<90 (total sleep time spent with SaO2<90%); ETCO2: Entidal CO2

*Comorbidities: diabetes, hypertension, coronary artery disease, heart failure, cancer, asthma, COPD/ emphysema and smoking pack years

© 2021 Pena Orbea C et al. *JAMA Network Open.*

**eTable 16.** Cox Proportional Hazard Models of Hospitalization and Death (N=1925)						
Independent variable	Model 1: Univariable		Model 2: Multivariable after adjustment of age, sex, race and BMI		Model 3: Multivariable after adjustment of age, sex, race, BMI, comorbidities*, healthcare system site	
	HR (95%CI)	p-value	HR (95%CI)	p-value	HR (95%CI)	p-value
Sleep Disordered Breathing Frequency Measure (AHI)						
CAI, 5 event/hr increment	1.10 (1.03 - 1.18)	*0.004*	1.03 (0.96 - 1.11)	0.42	1.03 (0.96 - 1.11)	0.45
OAI, 5 event/hr increment	1.01 (0.95 - 1.07)	0.81	0.97 (0.90 - 1.04)	0.37	0.98 (0.91 - 1.05)	0.60
Sleep-Related Hypoxia Measures						
Max ETCO2, 1 mmHg increment	1.01 (0.98 - 1.03)	0.65	1.00 (0.98 - 1.02)	0.82	1.00 (0.98 - 1.03)	0.74

Abbreviations: AHI: apnea hypopnea index; BMI; Body mass index; CAI: Central apnea index; OAI: Obstructive apnea index; HR: Hazards ratio; CI: Confidence Interval; TST<90 (total sleep time spent with SaO2<90%), ETCO2: Entidal CO2.
*Comorbidities: diabetes, hypertension, coronary artery disease, heart failure, cancer, asthma, COPD/ emphysema and smoking pack years.

© 2021 Pena Orbea C et al. *JAMA Network Open.*

Lost to follow-up and censored patients

eTable 17. Comparison by Lost to Follow-Up vs Censoring With Follow-Up in Time-to-Event Analysis of Hospitalization/Death

	Lost to follow-up (N=799)		Censoring (N=583)		
Factor	N	Statistics	N	Statistics	p-value
Age (yrs)	799	54.6 ± 13.7	583	52.0 ± 13.9	*<0.001*[a1]
Gender (Male)	799	413 (51.7)	583	271 (46.5)	0.06[c]
Race	799		583		*<0.001*[c]
White		575 (72.0)		352 (60.4)	
Black or African American		148 (18.5)		179 (30.7)	
Other		76 (9.5)		52 (8.9)	
Health Care system site	799		583		*<0.001*[c]
Ohio		792 (99.1)		547 (93.8)	
Florida		7 (0.88)		36 (6.2)	
Body mass index, kg/m^2	793	35.4 ± 8.4	574	36.4 ± 10.1	0.062[a2]
Comorbidities, n (%)					
Coronary Artery Disease	799	102 (12.8)	583	65 (11.1)	0.36[c]
Hypertension	799	380 (47.6)	583	348 (59.7)	*<0.001*[c]
Heart failure	799	55 (6.9)	583	46 (7.9)	0.48[c]
Asthma	799	159 (19.9)	583	173 (29.7)	*<0.001*[c]
COPD/emphysema	799	62 (7.8)	583	58 (9.9)	0.15[c]
Cancer	799	125 (15.6)	583	74 (12.7)	0.12[c]
Diabetes	799	183 (22.9)	583	162 (27.8)	*0.04*[c]
Smoking	799		583		*<0.001*[c]
No		611 (76.5)		320 (54.9)	
Current Smoker		30 (3.8)		48 (8.2)	
Former Smoker		158 (19.8)		215 (36.9)	
Smoking pack years	799		583		*<0.001*[b]
0.Never smoked		679 (85.0)		448 (76.8)	
0-10		52 (6.5)		53 (9.1)	
10-30		43 (5.4)		50 (8.6)	
30+		25 (3.1)		32 (5.5)	
Epworth Sleepiness Scale	745	9.6 ± 5.1	539	9.9 ± 5.4	0.42[a1]
Duration Sleep study before COVID test (years)	799	5.2 [2.8, 8.2]	583	4.1 [2.1, 7.3]	*<0.001*[b]
Sleep Procedure Type	741		536		0.88[c]
PSG		201 (27.1)		152 (28.4)	
Split		446 (60.2)		316 (59.0)	
Type III		94 (12.7)		68 (12.7)	
PAP use on COVID test	799	132 (16.5)	583	100 (17.2)	0.76[c]

© 2021 Pena Orbea C et al. *JAMA Network Open.*

	Lost to follow-up (N=799)		Censoring (N=583)		
Factor	N	Statistics	N	Statistics	p-value
Duration Sleep study before COVID test (years)	799	5.2 [2.8, 8.2]	583	4.1 [2.1, 7.3]	*<0.001*[b]
PAP use on COVID test	799	132 (16.5)	583	100 (17.2)	0.76[c]
Total Sleep Time, min	785	338.8 ± 81.4	569	337.8 ± 95.4	0.83[a2]
Apnea Hypopnea Index	799	15.8 [6.0, 39.4]	583	13.7 [5.3, 36.4]	0.16[b]
AHI categories:	799		583		0.20[b]
0-<5		152 (19.0)		125 (21.4)	
5-<15		231 (28.9)		175 (30.0)	
15-<30		159 (19.9)		107 (18.4)	
30+		257 (32.2)		176 (30.2)	
Central Apnea Index	423	0.00 [0.00, 0.20]	325	0.00 [0.00, 0.00]	*0.04*[b]
Obstructive Apnea Index	641	0.56 [0.00, 3.2]	468	0.51 [0.00, 2.7]	0.49[b]
% Sleep Time with SaO2<90%	774	1.4 [0.10, 12.1]	552	1.1 [0.00, 8.5]	0.16[b]
% Sleep Time with SaO2<90% dichotomized by median: > 1.8 %	774	361 (46.6)	552	242 (43.8)	0.31[c]
% Sleep Time with SaO2<90% categories by quartiles:	774		552		0.15[b]
0-0.1		219 (28.3)		175 (31.7)	
0.1-1.8		194 (25.1)		135 (24.5)	
1.8-12.8		176 (22.7)		126 (22.8)	
12.8-99.6		185 (23.9)		116 (21.0)	
Mean oxygen saturation	749	93.2 ± 2.6	548	93.4 ± 2.7	0.26[a1]
Minimum oxygen saturation	761	83.4 ± 8.1	559	83.8 ± 7.7	0.34[a1]
Maximum EtCO2 during sleep	202	49.2 ± 6.8	175	48.2 ± 6.2	0.12[a1]
Hospitalized	799	0 (0.00)	577	0 (0.00)	
Death	799		583		*<0.001*[c]
No		0 (0.00)		443 (76.0)	
Unknown		799 (100.0)		140 (24.0)	
WHO-7 COVID-19 Outcome, n (%)	799		583		0.99[b]
1.Not Hospitalized		799 (100.0)		583 (100.0)	

© 2021 Pena Orbea C et al. *JAMA Network Open.*

Factor	Lost to follow-up (N=799)		Censoring (N=583)		p-value
	N	Statistics	N	Statistics	
Days from symptom onset (or COVID19 test) to last follow-up (or hospitalization/death)	0	---	583	73.0 [13.0, 133.0]	

Statistics presented as Mean ± SD, Median [P25, P75], N (column %).
p-values: a1=t-test, a2=Satterthwaite t-test, b=Wilcoxon Rank Sum test, c=Pearson's chi-square test, d=Fisher's Exact test.

Results after multiple imputation

eTable 18. Ordinal Logistic Models of WHO Outcome Before and After Adjustment – After Multiple Imputation (N=1935)

Independent variable	Model 1: Univariable		Model 2: Multivariable after adjustment of age, sex, race and BMI		Model 3: Multivariable after adjustment of age, sex, race, BMI, comorbidities*, clinic site	
	OR (95%CI)	p-value	OR (95%CI)	p-value	OR (95%CI)	p-value
Sleep Disordered Breathing Frequency Measure (AHI)						
AHI, 5 event/hr increment	1.02 (1.01 - 1.04)	*0.006*	1.00 (0.98 - 1.02)	0.77	1.00 (0.98 - 1.02)	0.67
AHI⩾15 vs. <15	1.40 (1.15 - 1.70)	*<0.001*	1.05 (0.84 - 1.31)	0.68	1.02 (0.81 - 1.28)	0.88
AHI categories: 5-10 vs. 0-5	1.67 (1.21 - 2.30)	*0.002*	1.32 (0.93 - 1.86)	0.12	1.25 (0.88 - 1.77)	0.22
15-30 vs. 0-5	1.89 (1.35- 2.66)	*<0.001*	1.35 (0.94 - 1.96)	0.11	1.33 (0.91 - 1.94)	0.14
30+ vs. 0-5	2.01 (1.47 - 2.74)	*<0.001*	1.22 (0.86 - 1.75)	0.27	1.10 (0.76 - 1.59)	0.60
Sleep-Related Hypoxia Measures						
TST<90 by median: >1.8% vs. ⩽1.8%	1.87 (1.53- 2.29)	*<0.001*	1.51 (1.21 - 1.88)	*<0.001*	1.35 (1.08 - 1.69)	*0.008*
TST<90 quartiles: 0.1-1.8 vs. 0-0.1	1.58 (1.17 - 2.15)	*0.003*	1.22 (0.88 - 1.69)	0.24	1.20 (0.86 - 1.67)	0.28
1.8-12.8 vs. 0-0.1	2.27 (1.70 - 3.05)	*<0.001*	1.53 (1.11 - 2.10)	*0.010*	1.47 (1.06 - 2.04)	*0.02*
12.8-100 vs. 0-0.1	2.48 (1.86 - 3.32)	*<0.001*	1.88 (1.36 - 2.58)	*<0.001*	1.54 (1.11 - 2.14)	*0.01*
Mean SaO2, 5% increment	0.61 (0.52 - 0.73)	*<0.001*	0.67 (0.55 - 0.81)	*<0.001*	0.77 (0.63 - 0.94)	*0.01*
SaO2 nadir, 5% increment	0.86 (0.81 - 0.92)	*<0.001*	0.92 (0.86 - 0.98)	*0.010*	0.94 (0.87 - 1.00)	0.06

© 2021 Pena Orbea C et al. *JAMA Network Open.*

TST<90 , 5% increment	1.05 (1.03 - 1.07)	*<0.001*	1.04 (1.01 - 1.06)	*0.002*	1.02 (1.00 - 1.05)	0.09

Abbreviations: AHI: apnea hypopnea index; BMI; Body mass index; CAI: Central apnea index; OAI: Obstructive apnea index; HR: Hazards ratio; CI:Confidence Interval; TST<90 (total sleep time spent with SaO2<90%)
*Comorbidities: diabetes, hypertension, coronary artery disease, heart failure, cancer, asthma, COPD/ emphysema and smoking pack years

eTable 19. Cox Proportional Hazard Models of Hospitalization/Death Before and After Adjustment – After Multiple Imputation (N=1925)

Independent variable	Model 1: Univariable		Model 3: Multivariable after adjustment of age, sex, race and BMI		Model 5: Multivariable after adjustment of age, sex, race, BMI, comorbidities*, clinic site	
	HR (95%CI)	p-value	HR (95%CI)	p-value	HR (95%CI)	p-value
Sleep Disordered Breathing Frequency Measure (AHI)						
AHI, 5 event/hr increment	1.02 (1.01 - 1.03)	*0.006*	1.01 (0.99 - 1.02)	0.35	1.00 (0.99 - 1.02)	0.82
AHI⩾15 vs. <15	1.30 (1.10 - 1.54)	*0.003*	1.05 (0.88 - 1.26)	0.57	1.02 (0.85 - 1.22)	0.85
AHI categories: 5-10 vs. 0-5	1.51 (1.14 - 2.01)	*0.004*	1.22 (0.91 - 1.62)	0.18	1.17 (0.88 - 1.57)	0.28
15-30 vs. 0-5	1.62 (1.20 - 2.20)	*0.002*	1.19 (0.87 - 1.61)	0.27	1.15 (0.85 - 1.58)	0.37
30+ vs. 0-5	1.76 (1.33 - 2.32)	*<0.001*	1.23 (0.91 - 1.65)	0.17	1.13 (0.84 - 1.53)	0.41
Sleep-Related Hypoxia Measures						
TST<90 median: >1.8% vs. ⩽1.8%	1.62 (1.36 - 1.93)	*<0.001*	1.37 (1.14 - 1.65)	*<0.001*	1.29 (1.08 - 1.55)	*0.006*
TST<90 quartiles: 0.1-1.8 vs. 0-0.1	1.49 (1.13 - 1.95)	*0.004*	1.11 (0.84 - 1.46)	0.47	1.12 (0.85 -1.48)	0.41
1.8-12.8 vs. 0-0.1	1.96 (1.51 - 2.54)	*<0.001*	1.37 (1.04 - 1.79)	*0.02*	1.39 (1.06 - 1.82)	*0.02*
12.8-100 vs. 0-0.1	2.02 (1.57 - 2.61)	*<0.001*	1.56 (1.19 - 2.03)	*0.001*	1.38 (1.06 - 1.81)	*0.02*
Mean SaO2, 5% increment	0.71 (0.62 - 0.81)	*<0.001*	0.75 (0.64 - 0.87)	*<0.001*	0.83 (0.72 - 0.97)	*0.02*
SaO2 nadir, 5% increment	0.90 (0.86 - 0.94)	*<0.001*	0.94 (0.89 - 0.99)	*0.02*	0.95 (0.90 - 1.01)	0.08
TST<90, 5% increment	1.04 (1.02 - 1.05)	*<0.001*	1.03 (1.01 - 1.05)	*0.001*	1.02 (1.00 - 1.04)	*0.05*

Abbreviations: AHI: apnea hypopnea index; BMI; Body mass index; CAI: Central apnea index; OAI: Obstructive apnea index; HR: Hazards ratio; CI:Confidence Interval; TST<90 (total sleep time spent with SaO2<90%)
*Comorbidities: diabetes, hypertension, coronary artery disease, heart failure, cancer, asthma, COPD/ emphysema and smoking pack years

© 2021 Pena Orbea C et al. *JAMA Network Open.*

**eTable 20.** Cox Proportional Hazard Models of Hospitalization/Death Before and After Adjustment (N=1126, loss of follow-up excluded)						
Independent variable	Model 1: Univariable		Model 2: Multivariable after adjustment of age, sex, race and BMI		Model 3: Multivariable after adjustment of age, sex, race, BMI, comorbidities*, clinic site	
	HR (95%CI)	p-value	HR (95%CI)	p-value	HR (95%CI)	p-value
Sleep Disordered Breathing Frequency Measure (AHI)						
AHI, 5 event/hr increment	1.02 (1.01 - 1.04)	0.001	1.01 (1.00 - 1.03)	0.16	1.01 (0.99 - 1.02)	0.31
AHI⩾15 vs. <15	1.34 (1.13 - 1.59)	<0.001	1.10 (0.92 - 1.31)	0.29	1.11 (0.93 - 1.34)	0.24
AHI categories: 5-10 vs. 0-5	1.46 (1.10 - 1.94)	0.009	1.15 (0.86 - 1.53)	0.35	1.12 (0.84 - 1.51)	0.44
15-30 vs. 0-5	1.64 (1.21 - 2.21)	0.001	1.19 (0.87 - 1.61)	0.27	1.19 (0.87 - 1.62)	0.28
30+ vs. 0-5	1.78 (1.35 - 2.35)	<0.001	1.23 (0.92 - 1.65)	0.16	1.23 (0.91 - 1.66)	0.18
Sleep-Related Hypoxia Measures						
%Sleep Time SaO2<90% by median: >1.8% vs. ⩽1.8%	1.63 (1.37 - 1.95)	<0.001	1.31 (1.09 - 1.58)	0.004	1.25 (1.03 - 1.50)	0.02
%Sleep Time SaO2<90% by quartiles: 0.1-1.8 vs. 0-0.1	1.53 (1.16 - 2.01)	0.003	1.21 (0.91- 1.60)	0.18	1.25 (0.94 - 1.66)	0.12
1.8-12.8 vs. 0-0.1	1.93 (1.49 - 2.51)	<0.001	1.41 (1.07 - 1.85)	0.02	1.38 (1.05 - 1.82)	0.02
12.8-100 vs. 0-0.1	2.15 (1.65 - 2.79)	<0.001	1.54 (1.17 - 2.04)	0.002	1.47 (1.11 - 1.94)	0.008
SaO2 nadir, 5% increment	0.89 (0.85 - 0.94)	<0.001	0.92 (0.87 - 0.97)	0.003	0.92 (0.87 - 0.97)	0.003
SaO2 nadir, 5% increment	0.89 (0.85 - 0.94)	<0.001	0.92 (0.87 - 0.97)	0.003	0.92 (0.87 - 0.97)	0.003
Mean SaO2, 5% increment	0.70 (0.61 - 0.81)	<0.001	0.78 (0.66 - 0.91)	0.002	0.78 (0.66 - 0.91)	0.002

Abbreviations: AHI: apnea hypopnea index; BMI; Body mass index; CAI: Central apnea index; OAI: Obstructive apnea index; HR: Hazards ratio; CI: Confidence Interval; TST<90 (total sleep time spent with SaO2<90%)

*Comorbidities: diabetes, hypertension, coronary artery disease, heart failure, cancer, asthma, COPD/ emphysema and smoking pack years

© 2021 Pena Orbea C et al. *JAMA Network Open.*

eTable 21. Ordinal Logistic Models of WHO-7 Outcome (Stratify by COVID-19 Symptomatic or Asymptomatic)						
Independent variable	Model 1: Univariable		Model 2: Multivariable after adjustment of age, sex, race and BMI		Model 3: Multivariable after adjustment of age, sex, race, BMI, comorbidities*, clinic site	
	HR (95%CI)	p-value	HR (95%CI)	p-value	HR (95%CI)	p-value
COVID symptomatic (n=727)						
Sleep Disordered Breathing Frequency Measure (AHI)						
AHI, 5 event/hr increment	1.02 (1.00 - 1.04)	0.12	0.98 (0.96 - 1.01)	0.28	0.98 (0.95 - 1.01)	0.11
AHI⩾15 vs. <15	1.51 (1.13 - 2.02)	0.005	0.93 (0.68 - 1.29)	0.67	0.98 (0.70 - 1.36)	0.89
AHI categories: 5-10 vs. 0-5	2.07 (1.28 - 3.33)	0.003	1.53 (0.92 - 2.56)	0.10	1.45 (0.86 - 2.45)	0.16
15-30 vs. 0-5	2.44 (1.47 - 4.05)	<0.001	1.40 (0.81 - 2.43)	0.22	1.50 (0.86 - 2.61)	0.15
30+ vs. 0-5	2.48 (1.54 - 3.97)	<0.001	1.18 (0.69 - 2.02)	0.55	1.13 (0.65 - 1.96)	0.66
Sleep-Related Hypoxia Measures						
TST<90, median: >1.8% vs. ≤1.8%	2.23 (1.66 - 3.01)	<0.001	1.63 (1.17 - 2.26)	0.004	1.45 (1.03 - 2.02)	0.03
TST<90 quartiles: 0.1-1.8 vs. 0-0.1	1.73 (1.11 - 2.69)	0.02	1.36 (0.85 - 2.18)	0.20	1.32 (0.81 - 2.14)	0.26
1.8-12.8 vs. 0-0.1	2.63 (1.72 - 4.04)	<0.001	1.72 (1.08 - 2.75)	0.02	1.67 (1.03 - 2.71)	0.04
12.8-100 vs. 0-0.1	3.28 (2.13 - 5.05)	<0.001	2.23 (1.37 - 3.62)	0.001	1.73 (1.04 - 2.87)	0.04
Mean SaO2, 5% increment	0.59 (0.46 - 0.76)	<0.001	0.70 (0.52 - 0.94)	0.02	0.76 (0.56 - 1.04)	0.09
SaO2 nadir, 5% increment	0.84 (0.77 - 0.92)	<0.001	0.91 (0.82 - 1.01)	0.07	0.91 (0.82 - 1.01)	0.09
TST<90, 5% increment	1.06 (1.02 - 1.09)	0.002	1.04 (1.00 - 1.08)	0.04	1.02 (0.98 - 1.06)	0.33
COVID asymptomatic (n=1208)						
Sleep Disordered Breathing Frequency Measure (AHI)						
AHI, 5 event/hr increment	1.03 (1.01- 1.05)	0.01	1.02 (0.99- 1.04)	0.23	1.01 (0.98- 1.04)	0.48
AHI⩾15 vs. <15	1.37 (1.04- 1.80)	0.02	1.12 (0.82- 1.53)	0.49	1.02 (0.74- 1.41)	0.89
AHI categories: 5-10 vs. 0-5	1.37 (0.89- 2.13)	0.16	1.02 (0.63- 1.64)	0.94	1.07 (0.65- 1.75)	0.79
15-30 vs. 0-5	1.53 (0.96- 2.44)	0.07	1.12 (0.67- 1.87)	0.68	1.13 (0.66- 1.91)	0.66
30+ vs. 0-5	1.77 (1.17- 2.70)	0.007	1.14 (0.70- 1.86)	0.60	1.04 (0.63- 1.72)	0.89

© 2021 Pena Orbea C et al. *JAMA Network Open.*

Sleep-Related Hypoxia Measures						
%Sleep Time SaO2<90% by median: >1.8% vs. ⩽1.8%	1.93 (1.45 - 2.57)	<0.001	1.56 (1.14 - 2.14)	0.006	1.40 (1.01 - 1.94)	0.04
%Sleep Time SaO2<90% by quartiles:						
0.1-1.8 vs. 0-0.1	1.63 (1.04 - 2.56)	0.03	1.22 (0.76 - 1.98)	0.41	1.21 (0.74 - 1.97)	0.45
1.8-12.8 vs. 0-0.1	2.47 (1.61 - 3.78)	<0.001	1.65 (1.04 - 2.63)	0.04	1.59 (0.99 - 2.56)	0.06
12.8-100 vs. 0-0.1	2.57 (1.68 - 3.92)	<0.001	1.87 (1.17 - 2.97)	0.009	1.54 (0.95 - 2.50)	0.08
SaO2 nadir, 5% increment	0.86 (0.79 - 0.93)	<0.001	0.90 (0.82 - 0.99)	0.03	0.92 (0.83 - 1.01)	0.08
Mean SaO2, 5% increment	0.55 (0.43 - 0.70)	<0.001	0.60 (0.46 - 0.79)	<0.001	0.68 (0.51 - 0.90)	0.008
TST<90, 5% increment	1.06 (1.03 - 1.09)	<0.001	1.04 (1.01 - 1.08)	0.02	1.03 (0.99 - 1.06)	0.13

Abbreviations: AHI: apnea hypopnea index; BMI; Body mass index; OR: Odds ratio; CI: Confidence Interval; TST<90 (total sleep time spent with SaO2<90%)
*Comorbidities: diabetes, hypertension, coronary artery disease, heart failure, cancer, asthma, COPD/ emphysema and smoking pack years

© 2021 Pena Orbea C et al. *JAMA Network Open.*

eTable 22. Cox Proportional Hazard Models of Hospitalization and Death (Stratify by COVID Symptomatic or Asymptomatic)

Independent variable	Model 1: Univariable		Model 2: Multivariable after adjustment of age, sex, race and BMI		Model 3: Multivariable after adjustment of age, sex, race, BMI, comorbidities*, clinic site	
	HR (95%CI)	p-value	HR (95%CI)	p-value	HR (95%CI)	p-value
**COVID symptomatic (n=727)**						
**Sleep Disordered Breathing Frequency Measure (AHI)**						
AHI, 5 event/hr increment	1.01 (1.00 - 1.03)	0.12	1.00 (0.98 - 1.02)	0.82	1.00 (0.97- 1.02)	0.77
AHI≥15 vs. <15	1.30 (1.03 - 1.64)	0.03	0.98 (0.77 - 1.26)	0.89	1.04 (0.81 - 1.34)	0.75
AHI categories:						
5-10 vs. 0-5	1.78 (1.19 - 2.66)	0.005	1.34 (0.89 - 2.02)	0.16	1.29 (0.85 - 1.96)	0.23
15-30 vs. 0-5	1.83 (1.20 - 2.81)	0.005	1.22 (0.79 - 1.88)	0.38	1.25 (0.81 - 1.95)	0.32
30+ vs. 0-5	1.98 (1.33 - 2.95)	<0.001	1.23 (0.80 - 1.87)	0.35	1.26 (0.82 - 1.94)	0.30
**Sleep-Related Hypoxia Measures**						
TST<90, median: >1.8% vs. ≤1.8%	1.64 (1.29 - 2.09)	<0.001	1.34 (1.04 - 1.74)	0.02	1.27 (0.98 - 1.64)	0.07
TST<90 quartiles: 0.1-1.8 vs. 0-0.1	1.53 (1.06 - 2.20)	0.02	1.11 (0.76 - 1.62)	0.60	1.12 (0.76- 1.65)	0.57
1.8-12.8 vs. 0-0.1	1.90 (1.34 - 2.71)	<0.001	1.33 (0.91 - 1.93)	0.14	1.34 (0.92 - 1.97)	0.13
12.8-100 vs. 0-0.1	2.17 (1.53 - 3.08)	<0.001	1.56 (1.06 - 2.30)	0.02	1.37 (0.93 - 2.03)	0.11
Mean SaO2, 5% increment	0.78 (0.64 - 0.94)	0.01	0.86 (0.68 - 1.08)	0.19	0.89 (0.71 - 1.12)	0.31
SaO2 nadir, 5% increment	0.92 (0.86 - 0.98)	0.02	0.96 (0.89 - 1.04)	0.28	0.96 (0.89 - 1.03)	0.27
TST<90, 5% increment	1.03 (1.01 - 1.06)	0.01	1.03 (1.00 - 1.06)	0.05	1.02 (0.99 - 1.05)	0.19
**COVID asymptomatic (n=1208)**						
**Sleep Disordered Breathing Frequency Measure (AHI)**						
AHI, 5 event/hr increment	1.03 (1.01 - 1.04)	0.01	1.02 (0.99 - 1.04)	0.18	1.01 (0.98 -1.03)	0.53
AHI≥15 vs. <15	1.32 (1.03 - 1.70)	0.03	1.08 (0.83 - 1.42)	0.56	0.99 (0.75 - 1.30)	0.92
AHI categories: 5-10 vs. 0-5	1.28 (0.85 - 1.92)	0.23	0.98 (0.65 - 1.49)	0.94	1.08 (0.71 - 1.64)	0.72
15-30 vs. 0-5	1.42 (0.93 - 2.19)	0.11	1.02 (0.65 - 1.58)	0.94	1.05 (0.67 - 1.65)	0.83

© 2021 Pena Orbea C et al. *JAMA Network Open.*

30+ vs. 0-5	1.63 (1.11 - 2.39)	0.01	1.11 (0.73 - 1.69)	0.63	1.03 (0.67 - 1.58)	0.88
**Sleep-Related Hypoxia Measures**						
%Sleep Time SaO2<90% by median:						
>1.8% vs. ≤1.8%	1.76 (1.36 - 2.28)	<0.001	1.43 (1.09 - 1.88)	0.01	1.29 (0.98 - 1.70)	0.07
%Sleep Time SaO2<90% by quartiles:						
0.1-1.8 vs. 0-0.1	1.58 (1.04 - 2.40)	0.03	1.18 (0.77 - 1.80)	0.44	1.19 (0.78 -1.82)	0.43
1.8-12.8 vs. 0-0.1	2.26 (1.52 - 3.35)	<0.001	1.54 (1.02 - 2.31)	0.04	1.49 (0.99 - 2.24)	0.06
12.8-100 vs. 0-0.1	2.23 (1.51 - 3.30)	<0.001	1.62 (1.08 - 2.44)	0.02	1.37 (0.91 - 2.08)	0.13
SaO2 nadir, 5% increment	1.58 (1.04 - 2.40)	0.03	1.18 (0.77-1.80)	0.44	1.19 (0.78 - 1.82)	0.43
Mean SaO2, 5% increment	0.62 (0.51 - 0.75)	<0.001	0.67 (0.54 - 0.83)	<0.001	0.77 (0.62 - 0.96)	0.02
TST<90, 5% increment	1.05 (1.02 - 1.07)	<0.001	1.03 (1.00 - 1.06)	0.02	1.02 (0.99 - 1.05)	0.26

Abbreviations: AHI: apnea hypopnea index; BMI; Body mass index; OR: Odds ratio; CI: Confidence Interval; TST<90 (total sleep time spent with SaO2<90%)

*Comorbidities: diabetes, hypertension, coronary artery disease, heart failure, cancer, asthma, COPD/ emphysema and smoking pack years

© 2021 Pena Orbea C et al. *JAMA Network Open.*

eReferences

1. Zhou Y, Hou Y, Shen J, Huang Y, Martin W, Cheng F. Network-based drug repurposing for novel coronavirus 2019-nCoV/SARS-CoV-2. *Cell Discovery*. 2020;6(1). doi:10.1038/s41421-020-0153-3
2. Milinovich A, Kattan MW. Extracting and utilizing electronic health data from Epic for research. *Annals of Translational Medicine*. 2018;6(3):42-42. doi:10.21037/atm.2018.01.13
3. Harris PA, Taylor R, Minor BL, et al. The REDCap consortium: Building an international community of software platform partners. *Journal of Biomedical Informatics*. 2019;95:103208. doi:10.1016/j.jbi.2019.103208
4. Harris PA, Taylor R, Thielke R, Payne J, Gonzalez N, Conde JG. Research electronic data capture (REDCap)-A metadata-driven methodology and workflow process for providing translational research informatics support. *Journal of Biomedical Informatics*. 2009;42(2):377-381. doi:10.1016/j.jbi.2008.08.010
5. Muraki I, Tanigawa T, Yamagishi K, et al. Nocturnal intermittent hypoxia and C reactive protein among middle-aged community residents: A cross-sectional survey. *Thorax*. 2010;65(6):523-527. doi:10.1136/thx.2009.128744
6. Daher A, Balfanz P, Aetou M, et al. Clinical course of COVID-19 patients needing supplemental oxygen outside the intensive care unit. *Scientific Reports*. 2021;11(1):2256. doi:10.1038/s41598-021-81444-9

© 2021 Pena Orbea C et al. *JAMA Network Open.*
